# IL‐21‐IgFc immunotherapy alters transcriptional landscape of lymph node cells leading to enhanced flu vaccine response in aging and SIV infection

**DOI:** 10.1111/acel.13984

**Published:** 2023-09-15

**Authors:** Suresh Pallikkuth, Daniel Kvistad, Tirupataiah Sirupangi, Alexander Kizhner, Rajendra Pahwa, Mark J. Cameron, Brian Richardson, Sion Williams, Ana Ayupe, Marissa Brooks, Constantinos Petrovas, Francois Villinger, Savita Pahwa

**Affiliations:** ^1^ Department of Microbiology and Immunology University of Miami School of Medicine Miami Florida USA; ^2^ New Iberia Research Center and Department of Biology University of Louisiana at Lafayette New Iberia Louisiana USA; ^3^ Department of Quantitative and Population Health Sciences Case Western Reserve University Cleveland Ohio USA; ^4^ Department of Neurology, Onco‐Genomics Shared Resource, Sylvester Comprehensive Cancer Center University of Miami School of Medicine Miami Florida USA; ^5^ Onco‐Genomics Shared Resource, Sylvester Comprehensive Cancer Center University of Miami School of Medicine Miami Florida USA; ^6^ Tissue Analysis Core, Immunology Laboratory, Vaccine Research Center NIAID, NIH Bethesda Maryland USA; ^7^ Department of Laboratory Medicine and Pathology Institute of Pathology, Lausanne University Hospital and Lausanne University Lausanne Switzerland

**Keywords:** aging and immune response, aging and SIV, IL‐21 and Tfh cells, IL‐21 and vaccine response, immunomodulation in aging

## Abstract

Aging people living with HIV (PWH) frequently manifest impaired antibody (Ab) responses to seasonal flu vaccination which has been attributed to ongoing inflammation and immune activation. We have recently reported a similar scenario in old simian immunodeficiency virus (SIV) infected rhesus macaques (RM) with controlled viremia and have been able to compensate for this deficiency by immunotherapy with interleukin (IL)‐21‐IgFc. To understand the underlying mechanisms of IL‐21‐induced immunomodulation leading to enhanced flu vaccine response in aging and SIV, we have investigated draining lymph node (LN) cells of IL‐21‐treated and ‐untreated animals at postvaccination. We observed IL‐21‐induced proliferation of flu‐specific LN memory CD4 T cells, expansion of B cells expressing IL‐21 receptor (IL‐21R), and modest expansion of T follicular helper cells (Tfh) co‐expressing T‐cell immunoreceptor with Ig and ITIM domains (TIGIT) and DNAX accessory molecule (DNAM‐1). Transcriptional analysis of LN cells of IL‐21‐treated animals revealed significant inhibition of germinal center (GC) Tfh and B‐cell interferon signaling pathways along with enhanced B‐cell development and antigen presentation pathways. We conclude that IL‐21 treatment at the time of flu vaccination in aging SIV‐infected animals modulates the inductive LN GC activity, to reverse SIV‐associated LN Tfh and B‐cell dysfunction. IL‐21 is a potential candidate molecule for immunotherapy to enhance flu vaccine responses in aging PWH who have deficient antibody responses.

## Graphical abstract

IL‐21‐IgFC enhances influenza vaccine responses in aged macaques with suppresses SIV infection.

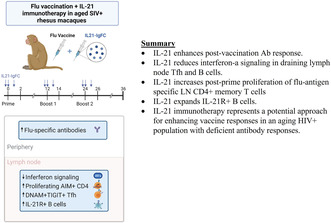

**Abbreviations**
Ab: antibody
DNAM‐1: DNAX accessory molecule‐1
GC: germinal center
ICOS: inducible costimulator
Ig: immunoglobulin
IL: interleukin
IL‐21R: interleukin 21 receptor
LN: lymphnode
PWH: people with HIV
SIV: simian immunodeficiency virus
Tfh: T follicular helper cells
TIGIT: T‐cell immunoglobulin and ITIM domain

## 1 INTRODUCTION

Due to waning immunity in old age, individuals who are 65 years of age or older are at greater risk for hospitalization and death due to flu infection (Grohskopf et al., 2021; Pallikkuth et al., 2018). The risk of seasonal flu disease burden and serious flu‐related complications is even greater in people living with HIV (PWH) because of premature immunological aging HIV Among People Aged 50 and Over|Age|HIV by Group|HIV/AIDS, n.d.). Seasonal flu vaccination is used for prevention of flu infection, however clinical protection is variable from year to year, and lower in the elderly and PWH, underscoring the need for effective approaches to improve vaccine‐induced immunity among the growing demographic of PWH over the age of 50.

Humoral immunity to vaccines is induced in germinal center (GC) reactions within lymph node (LN) follicles (Crotty, 2019; Juno & Hill, 2022). Within LN GCs, specialized CD4 T cells known as T follicular helper (Tfh) cells expressing lymphoid homing receptor CXC chemokine receptor 5 (CXCR5) provide nonredundant help to cognate antigen‐primed B cells in the form of receptor‐ligand interactions (including CD40/CD40L and ICOS/ICOSL) and cytokine secretion (including IL‐21 and IL‐4) resulting in B‐cell differentiation, proliferation, and Ab secretion (Crotty, 2019; Juno & Hill, 2022; Muppidi & Klein, 2020). IL‐21, a pleiotropic γ‐chain signaling cytokine is produced by Tfh cells leading to the formation and maintenance of LN GC reactions (Crotty, 2011) and plays a vital role in the dynamics, quality, quantity, class, and final outcome of a Tfh‐mediated humoral immune response (Crotty, 2011; Tangye & Ma, 2020).

In HIV infection, decreased ability of HIV specific CD4 T cells to produce IL‐21 has been reported (Pallikkuth et al., 2019). Moreover, plasma IL‐21 levels correlated directly with CD4 count and inversely with HIV viral load and the IL‐21 levels were higher in elite controllers (EC) of HIV infection, supporting a role of IL‐21 in viral control (Iannello et al., 2010). In EC, IL‐21 production by HIV‐specific CD4 T cells is regulated through immunometabolism involving strong autophagy‐mediated proteolysis (Loucif et al., 2022). In addition, the therapeutic utility of IL‐21 has been investigated in a number of human malignant disorders such as metastatic renal cell carcinoma, metastatic melanoma, and relapsed/refractory indolent non‐Hodgkin's lymphoma, with demonstrable antitumor activity (reviewed in Eivary et al., 2023).

Chronic inflammation and immune activation associated with aging and HIV infection contribute to impaired immunity to seasonal flu vaccine‐induced Ab response (de Armas et al., 2017; Pallikkuth et al., 2018). Other factors that could influence the vaccine response include chronic infection such as *Schistosoma mansoni* (Muir et al., 2023), latent CMV infection (Royston et al., 2021), and transcriptional state of the innate immune cells (Fourati et al., 2022). Given the critical role of Tfh cells in eliciting a robust flu vaccine‐induced Ab response (Crotty, 2019; Juno & Hill, 2022), we and others have demonstrated that both HIV‐negative older (≥60 years) individuals and PWH at all ages exhibit weak flu vaccine‐induced Ab responses which associate with low postvaccination peripheral (p)Tfh levels and low IL‐21 production by pTfh cells compared to young person without HIV (PWoH) (George et al., 2015; Koutsakos et al., 2019). Additionally, LN biopsies from flu‐vaccinated PWH revealed that prevaccination frequencies of Tfh cells predict postvaccination Ab titers to influenza B antigens, and that PWH display altered immune cell follicular dynamics including the decline of Tfh cell frequencies following flu vaccination compared to PWoH (Moysi et al., 2018).

Recent studies have shown strong evidence for the reliability of the rhesus macaque model to accurately portray the pathophysiology and immunological perturbations associated with natural aging as well as chronic ART‐controlled HIV infection in humans. Older animals showed inflammaging characterized by increase in multiple biomarkers of inflammation along with alterations in the peripheral and lymphoid immune cell compartment similar to human aging, supporting the utility of this NHP model in aging immunity studies (Pallikkuth et al., 2021; Shankwitz et al., 2020). In the B‐cell compartment, the frequencies of activated memory B cells progressively increased with SIV infection and aging, and inversely correlated with the magnitude of SIV‐specific IgG responses, along with impaired maturation of anti‐SIV antibody avidity, as seen with HIV‐1 infection (Chang et al., 2017). We recently demonstrated that IL‐21 immunotherapy enhances flu vaccine‐induced Ab responses in aging simian immunodeficiency virus (SIV) infected ART‐controlled rhesus macaques (RM) resulting in increased LN GC activity with expansion of TIGIT+ pTfh in circulation (Kvistad et al., 2021). The present study was aimed at gaining mechanistic insights into IL‐21‐induced immunomodulation of Tfh and B cells at the inductive site of LN and investigated the draining LN mononuclear cell (LNMC) suspensions at 14 days after flu vaccination in IL‐21‐treated and ‐untreated old SIV+ RMs along with the flu vaccine response.

## 2 RESULTS

### 2.1 Study design

As we recently described (Kvistad et al., 2021), Old SIV+ IL‐21‐treated and ‐untreated animals were given flu vaccine in a prime/boost 1 (B1)/boost 2 (B2) immunization schedule (Figure S1a). Antibody responses among IL‐21‐treated old SIV+ animals measured as fold change (FC) from the pre‐prime baseline timepoint were significantly increased at Day 14 post‐first boost (B1, *p* = 0.015) (Figure S1b), but not Day 14 post‐second boost (B2, Figure S1c). At Day 84 post‐B2 Ab levels were significantly higher in the IL‐21‐treated animals owing to the sustained response (B2, *p* = 0.017) while the untreated animals showed further decline in the FC Ab response (Figure S1d). To further investigate the adjuvant effects of IL‐21 on Tfh and B cells, we examined the LNMC. Samples at Day 14 post prime (LNMC), Day 14 post‐B1 (LNMC and PBMC) and Day 14 post‐B2 (LNMC and PBMC) were stimulated with a mix of H1N1/H3N2 HA flu antigens (subunits of the flu vaccine), SEB (positive control), or left unstimulated (negative control) (Figure S1e). Additionally, 10× single cell (sc)‐RNAseq was performed on Day 14 post‐B1 LN cells from both the media and flu‐stimulated conditions of two animals from each group (IL‐21‐treated and ‐untreated).

### 2.2 DNAM/TIGIT expression is markedly heterogeneous among LN CD4 T cell populations

**FIGURE 1:**
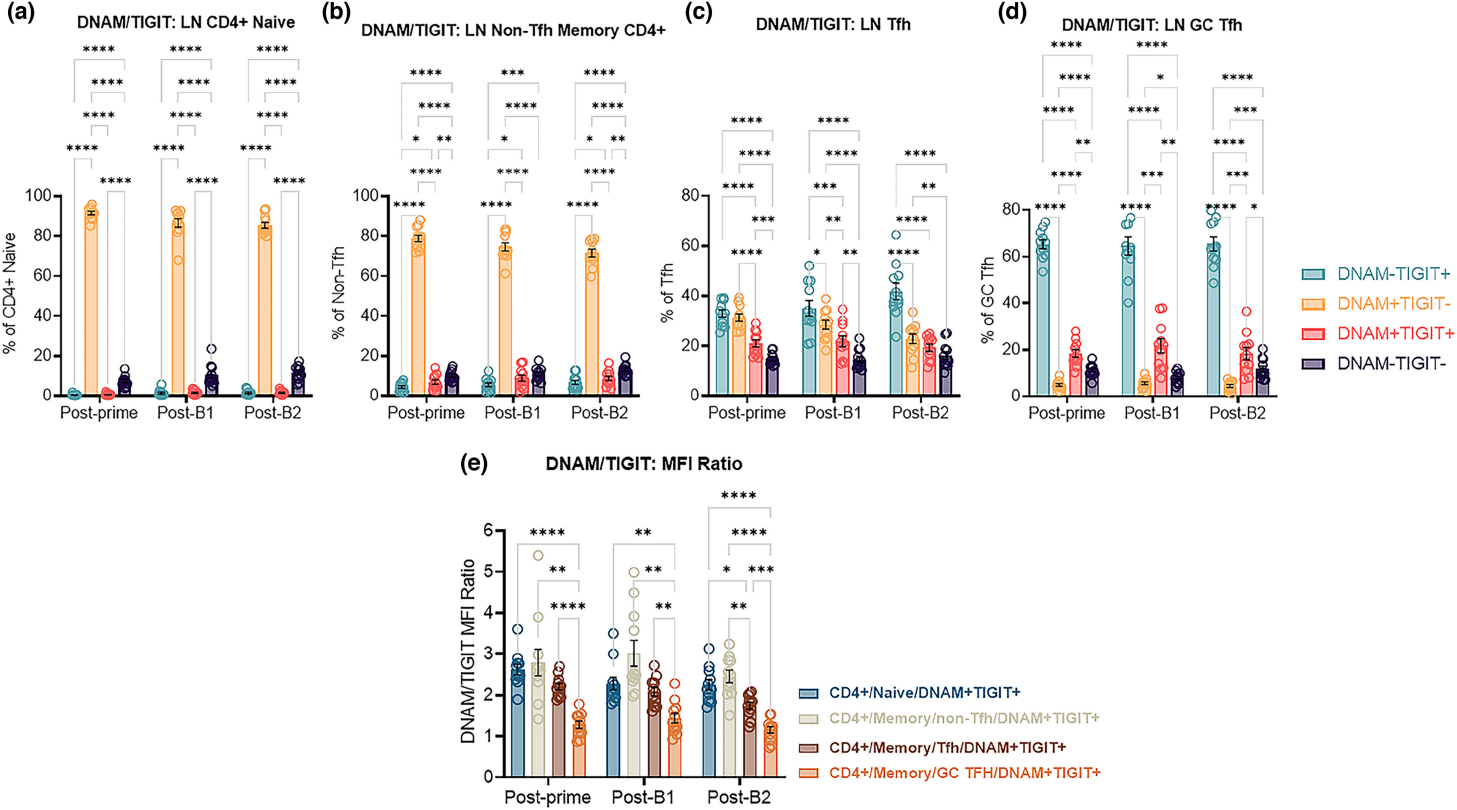
TIGIT is highly expressed on LN Tfh and GC Tfh populations: Ex‐vivo day 14 post‐prime, post‐B1 and post‐B2 frequencies of DNAM−TIGIT+, DNAM+TIGIT−, DNAM+TIGIT+, and DNAM−TIGIT− on (a) LN CD4+ naïve (CD4+CD95−), (b) LN non‐Tfh memory CD4^+^ T cells (CD4+CD95+CXCR5−PD1−), (c) LN Tfh (CD4+CD95+CXCR5+PD1+), and (d) LN GC Tfh (CD4+CD95+CXCR5++PD1++). (e) DNAM/TIGIT MFI ratio on ex‐vivo DNAM+TIGIT+ LN CD4 subsets on Day 14 post‐prime, post‐B1 and post‐B2. Old SIV+ IL‐21+ (*n* = 7) and old SIV+ IL‐21− (*n* = 4) animals were combined for all comparisons due to lack of significant difference between groups. Data are displayed as mean + SEM. Statistical analysis by two‐way anova with multiple comparison corrections by Benjamini, Krieger and Yekutieli. *≤0.05; **≤0.01; ***≤0.001; ****≤0.0001.

We sought to investigate the expression on LN Tfh populations of both TIGIT and the receptor DNAM‐1 (herein referred to as DNAM), which bind the same ligand, CD155 (expressed on APCs, Alteber et al., 2021). Upon receptor‐ligand binding on T cells, DNAM evokes a stimulatory signal whereas TIGIT induces an inhibitory signal, indicating the immunoregulatory role of these markers. Moreover, TIGIT+ pTfh exhibited superior B‐cell helper functions, promoting greater B‐cell differentiation and antibody production than TIGIT− pTfh (Godefroy et al., 2015). As there is little knowledge of TIGIT and DNAM among LN CD4 T‐cell subsets, we investigated the TIGIT/DNAM co‐expression on ex‐vivo CD4 Naïve, non‐Tfh memory, Tfh (CXCR5+PD1+), and bona‐fide GC Tfh (CXCR5++PD1++) cells. Surprisingly TIGIT and DNAM expression was very heterogeneous between LN CD4 subsets; with CD4 Naïve and non‐Tfh memory cells predominantly (~80%–90%) DNAM+TIGIT− at all three postvaccination timepoints (Figure 1a,b), with no differences between IL‐21‐treated and ‐untreated animals (data not shown). Next, we examined LN non‐GC Tfh (LN‐Tfh) cells which displayed a much more diverse profile with approximately 30%–40% DNAM−TIGIT+, ~25%–30% DNAM+TIGIT−, ~20% DNAM+TIGIT+, and ~15% DNAM−TIGIT− cells (Figure 1c), with no differences between IL‐21‐treated and ‐untreated animals (data not shown). LN GC Tfh cells were markedly different from LN Tfh cells with ~65% being DNAM−TIGIT+, ~5% DNAM+TIGIT−, ~20% DNAM+TIGIT+, and ~10% DNAM−TIGIT− (Figure 1d), with no differences between IL‐21‐treated and ‐untreated (data not shown). Together, these data illustrate the stark differences in DNAM and TIGIT expression across LN CD4 T cell subsets, with LN Tfh and GC Tfh cells being enriched for TIGIT single‐positive and TIGIT/DNAM double‐positive subsets as compared to the predominantly DNAM single expression in CD4 naïve and non‐Tfh cells (Figure 1a–d).

We next compared the ratio of DNAM/TIGIT median fluorescence intensity (MFI) between LN naïve CD4, non‐Tfh memory CD4, LN Tfh (CXCR5+PD‐1+) as well as GC Tfh (CXCR5++PD1++) at all three postvaccine timepoints for IL‐21‐treated and ‐untreated animals (Figure 1e). We observed a DNAM/TIGIT MFI ratio of ~3:1 on both naïve and non‐Tfh memory CD4 T cells, while LN Tfh had a ratio of ~2:1 and GC Tfh had a ratio of ~1:1 (Figure 1e). Together, these data show that the DNAM/TIGIT ratio is highest among DNAM+TIGIT+ LN CD4 naïve and non‐Tfh, and progressively lowers among DNAM+TIGIT+ LN Tfh and GC Tfh, implicating a shift in the balance of activating and inhibitory signals these cells receive during their differentiation, possibly following cell–cell interaction with CD155 expressing APCs, including B cells, indicating a potential immunoregulatory effect.

### 2.3 Flu antigen‐specific LN and PBMC Tfh populations increase postvaccination and peak at Day 14 post‐B2

In order to understand how IL‐21 immunotherapy impacts the kinetics of flu antigen‐specific LN CD4 T‐cell populations (Figure S2a,b), we assessed the frequencies of activation induced molecules (AIM)+ (CD25+OX40+) CD4 T‐cell populations (Figure S3a–e). Compared to the unstimulated condition, frequencies of flu‐stimulated AIM+CXCR5+ memory CD4 T cells (AIM+CXCR5+ memory CD4) and AIM+ LN Tfh increased significantly at post‐B1 and at post‐B2 in both IL‐21‐treated and ‐untreated animals (Figure S3a,b). Among AIM+ GC Tfh, only IL‐21‐treated animals displayed a significant increase in frequency at post‐B2 (*p* = 0.001) compared to unstimulated condition (Figure S3c). In post‐B1 PBMC, IL‐21 treated animals displayed a significantly higher frequency of circulating flu‐specific AIM+CXCR5+ memory CD4 T cells while at post‐B2, both IL‐21‐treated and ‐untreated animals had a significant increase in circulating AIM+ CXCR5+ memory CD4 T cells compared to unstimulated conditions ((Figure S3d,e). Furthermore, within the flu‐stimulated condition both circulating AIM+ CXCR5+ memory CD4 T cells (Figure S3d) and AIM+ Tfh (Figure S3e) were significantly increased between post‐B1 and post‐B2 timepoints in both IL‐21‐treated and ‐untreated animals. Together, the kinetics of AIM+ PBMC populations parallel our observations of significantly higher post‐B1 Ab responses in IL‐21‐treated animals. Taken together, these data indicate that IL‐21 appears not to have significantly altered the quantity of flu‐specific Tfh populations in draining LNs, but did alter the quantity in circulation, perhaps suggesting that Day 14 postvaccination measurements of antigen‐specific pTfh in circulation may more closely resemble the kinetics of flu vaccine‐induced Ab responses.

### 2.4 Flu antigen‐specific LN Tfh and GC Tfh are enriched for DNAM−TIGIT+ and DNAM+TIGIT+ expression

**FIGURE 2:**
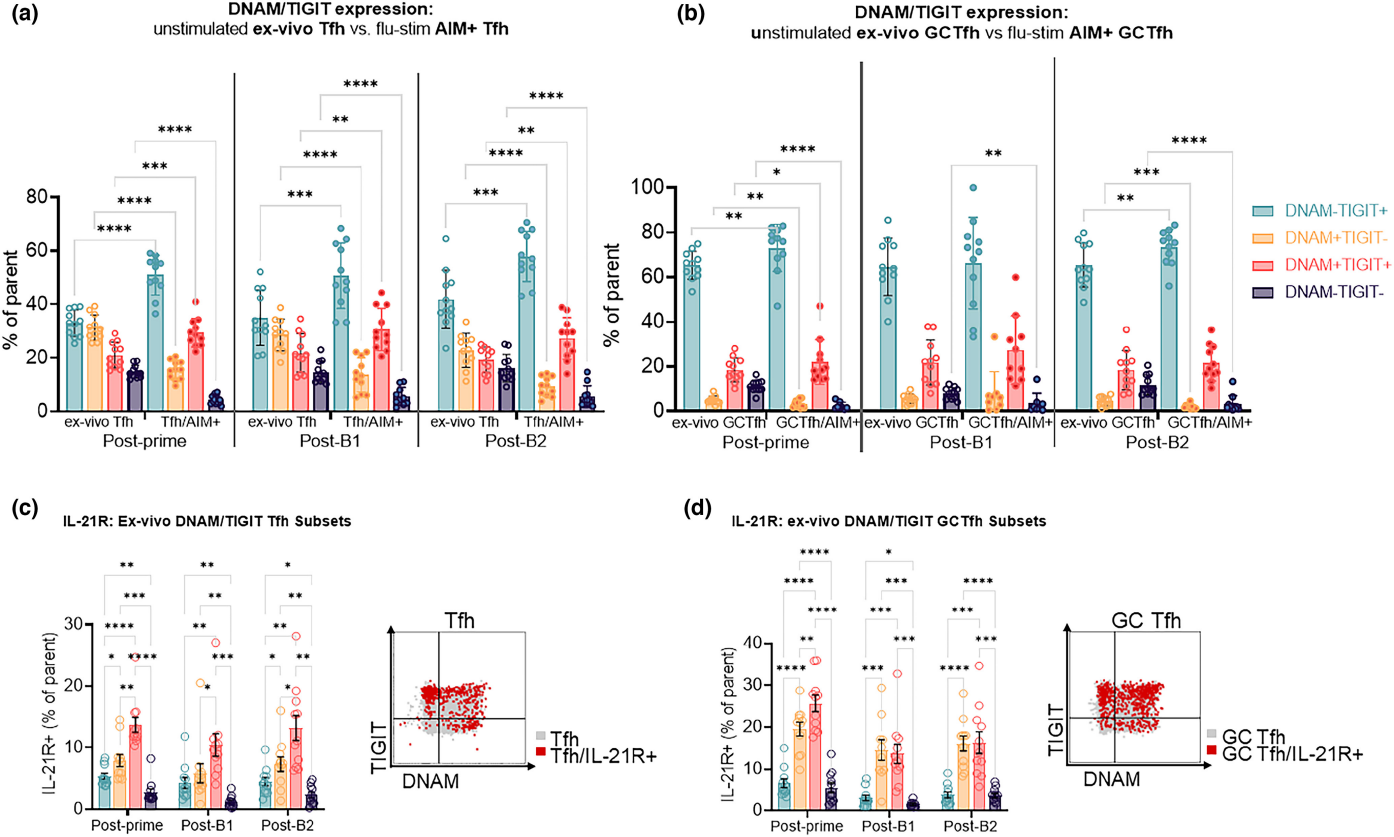
AIM+ LN Tfh and GC Tfh are enriched for TIGIT expression: (a) Comparison of DNAM/TIGIT‐negative, single‐positive, and double‐positive subsets between the total LN Tfh population from the ex‐vivo unstimulated condition and the AIM+ (OX40+CD25+) LN Tfh population from the flu‐stimulated condition at all three Day 14 postvaccination timepoints. (b) Comparison of DNAM/TIGIT‐negative, single‐positive, and double‐positive subsets between the total LN GC Tfh population from the ex‐vivo unstimulated condition and the AIM+ (OX40+CD25+) LN Tfh population form the flu‐stimulated condition at all Day 14 postvaccination timepoints. Frequency of IL‐21R+ cells among DNAM/TIGIT‐negative, single‐positive, and double‐positive subsets and corresponding representative IL‐21R+ cell overlay flow plot for (c) LN Tfh and (d) LN GC Tfh cells at all three Day 14 postvaccination timepoints. Data are shown for both treatment groups combined and displayed as mean + SEM. Statistical analysis by two‐way anova with multiple comparison corrections by Benjamini, Krieger, and Yekutieli. *≤0.05; **≤0.01; ***≤0.001; ****≤0.0001.

Next, we compared DNAM/TIGIT expression in LN Tfh (Figure 2a) and GC Tfh (Figure 2b) populations between ex‐vivo total LN Tfh/GC Tfh and flu‐stimulated AIM+ populations at all timepoints. AIM+ Tfh had significantly higher frequencies of DNAM−TIGIT+ and DNAM+TIGIT+ and significantly lower frequencies of DNAM+TIGIT− and DNAM−TIGIT− compared to ex‐vivo LN Tfh (Figure 2a). Similarly, AIM+ GC Tfh were significantly enriched for DNAM−TIGIT+ DNAM+TIGIT+ populations compared to ex‐vivo GC Tfh (Figure 2b). The frequencies of DNAM/TIGIT expressing subsets were compared between ex‐vivo LN Tfh/GC Tfh and flu stimulated total LN Tfh/GC Tfh and starkly less differences were observed (Figure S4a,b). We also compared the IL‐21‐treated and ‐untreated ex‐vivo LN Tfh/GC Tfh (Figure S4c,d) and flu‐stimulated AIM+ Tfh/GC Tfh (Figure S4e,f) and observed minimal differences between groups (Figure S4c–f). Altogether these data show that AIM+ flu‐specific LN Tfh are enriched for DNAM−TIGIT+ and DNAM+TIGIT+ cell subsets compared to total Tfh, potentially representing a phenotypic shift associated with the antigen‐induced activation specific to LN Tfh cell populations.

We next investigated the IL‐21R expression on combinations of single‐ and double‐positive TIGIT and DNAM expressing LN Tfh and GC Tfh across all animals, regardless of IL‐21 treatment (Figure 2c,d). At all timepoints, DNAM+ TIGIT+ and DNAM+TIGIT− Tfh and GC Tfh exhibited the highest and second highest IL‐21R expression, respectively, compared to DNAM−TIGIT− or DNAM−TIGIT+ Tfh and GC Tfh (Figure 2c,d). Although IL‐21R expression tended to be higher in IL‐21‐treated animals, it was not significantly different from untreated animals (Figure S5a–c). Together, high IL‐21R expression among DNAM+ LN Tfh and GC Tfh suggests that DNAM expressing LN Tfh may be more responsive to IL‐21 immunotherapy and comprise a novel subset of IL‐21 responding Tfh capable of providing strong B cell help to facilitate vaccine‐induced Ab production.

### 2.5 IL‐21 immunomodulation of vaccine responses associates with ex‐vivo DNAM+TIGIT+ LN Tfh and IL‐21R+ B cells

**FIGURE 3:**
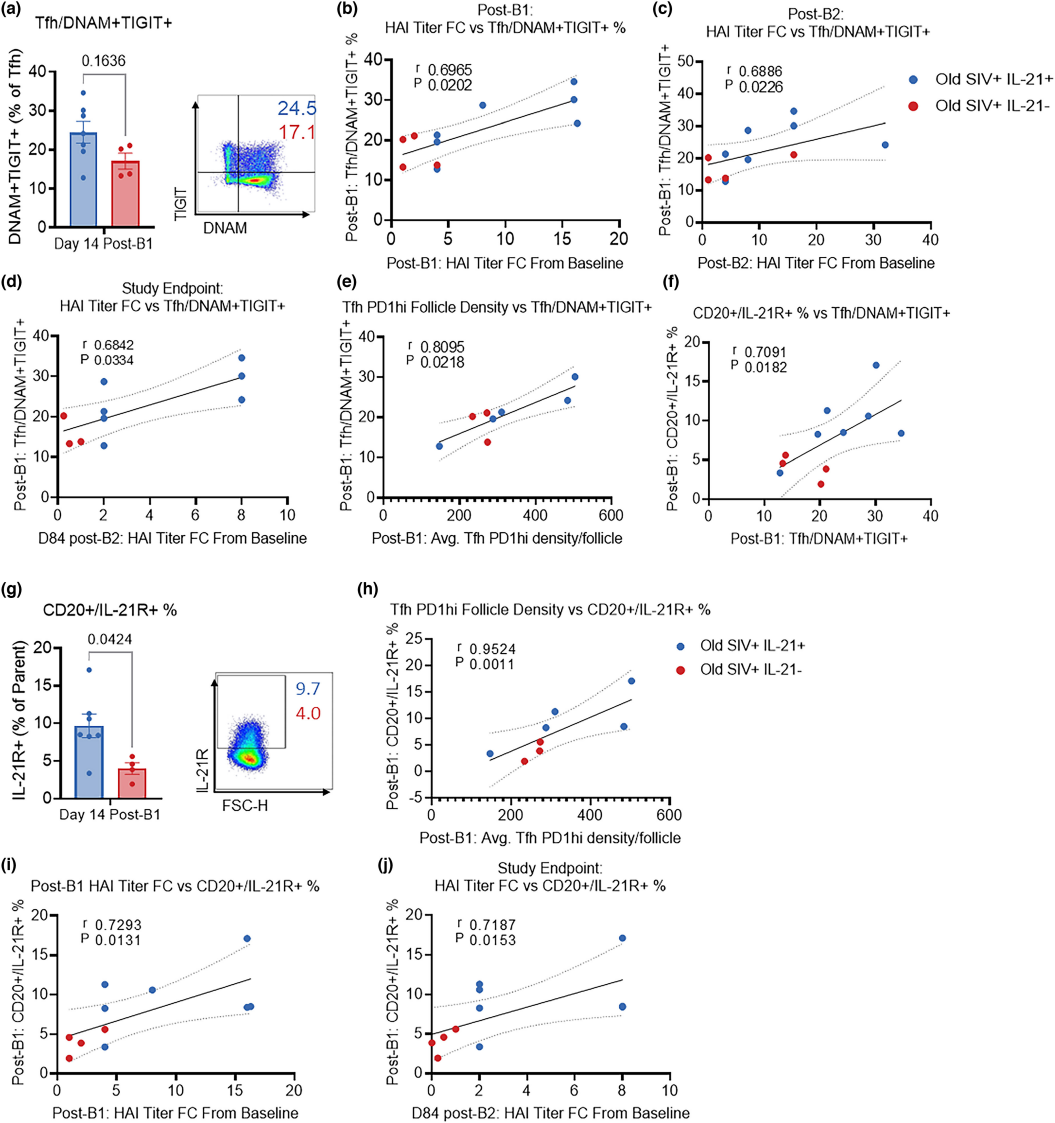
Ex‐vivo LN Tfh co‐expressing DNAM and TIGIT associate with vaccine response and ex‐vivo LN IL‐21R+ B cells: (a), Bar graph showing Day 14 post‐B1 frequency of ex‐vivo DNAM+TIGIT+ LN Tfh and the representative flow plot. Spearman correlations between the Day 14 post‐B1 frequency of ex‐vivo DNAM+TIGIT+ LN Tfh cells and (b) Day 14 post‐B1 HAI titer FC from baseline, (c) Day 14 post‐B2 HAI titer FC from baseline, (d) Day 84 post‐B2 HAI titer FC from baseline (due to sample non‐availability, one missing animal from control group), (e) average Day 14 post‐B1 Tfh (CD4+PD1+) density per draining LN follicle area mm^2^, and (f) Day 14 post‐B1 frequency of ex‐vivo IL‐21R+ LN B cells. (g) Day 14 post‐B1 frequency of ex‐vivo IL‐21R+ LN B cells (Lin‐CD20+) and the representative flow plot. Spearman correlations between the Day 14 post‐B1 frequency of ex‐vivo IL‐21R+ LN B cells and (h) average Day 14 post‐B1 Tfh density per draining LN follicle area mm^2^, (i) Day 14 post‐B1 HAI titer FC from baseline and (j) Day 84 post‐B2 HAI titer FC from baseline. Due to sample quality and availability, analysis of draining LN tissue was performed with 5/8 animals from old SIV+IL‐21+, and 3/4 animals from old SIV+IL‐21− groups. Data are displayed as mean ± SEM, blue dots represent old SIV+IL‐21+ animals (due to missing sample from one animal, *n* = 7), while red circles represent old SIV+IL‐21− animals (*n* = 4). Data analyzed by two‐tailed Mann–Whitney test.

We next investigated the frequency of ex‐vivo DNAM+TIGIT+ Tfh at day 14 post‐B1 (Figure 3a), the timepoint at which Ab responses among IL‐21‐treated animals were significantly higher. Frequencies of DNAM+TIGIT+ LN‐Tfh at Day 14 post‐B1 trended higher in the IL‐21‐treated compared to untreated animals (Figure 3a). Interestingly, the frequency of ex‐vivo Day 14 post‐B1 TIGIT+DNAM+ LN Tfh correlated with the FC in HAI titer from baseline to Day 14 post‐B1 (*r* = 0.6965; *p* = 0.0202, Figure 3b), Day 14 post‐B2 (*r* = 0.6886; *p* = 0.0226, Figure 3c) and Day 84 post‐B2 study endpoint (*r* = 0.6801; *p* = 0.0334, Figure 3d) as well as to post‐B1 LN Tfh density/follicle (*r* = 0.8096; *p* = 0.0218, Figure 3e). We did not find any association with other LN subsets (DNAM−TIGIT+, DNAM+TIGIT−, and DNAM−TIGIT−) with FC in HAI titer from baseline at post‐boost1, at post‐boost 2, and at study endpoint (data not shown). The frequency of DNAM+TIGIT+ LN Tfh correlated with the frequency of ex‐vivo post‐B1 LN B cells expressing IL‐21R (0.7091; *p* = 0.0182, Figure 3f). Post‐B1 frequencies of IL‐21R+ B cells were also higher in IL‐21‐treated animals (*p* = 0.0424, Figure 3g) which significantly correlated with FC HAI titer from baseline to Day 14 post‐B1 (*r* = 0.7293; *p* = 0.0131, Figure 3i), LN Tfh density/follicle on Day 14 post‐B1 (*r* = 0.9524; *p* = 0.0011, Figure 3h) and FC in HAI titer from baseline to Day 84 post‐B2 (*r* = 0.7187; *p* = 0.0153, Figure 3j). Analysis of total LN B cells, naïve, resting memory (RM), and activated memory (AM) B cells were not significantly different between IL‐21‐treated versus ‐untreated groups (Figure S6a–d). In agreement with our previous report (Kvistad et al., 2021), a trend of higher frequencies of activated memory (AM) B cells in IL‐21‐treated animals at each time point indicating an effect of IL‐21 in enhancing the B‐cell activation and differentiation. The frequencies of AM B cells at Day 14 post‐boost 1 correlated with the FC HAI titer from baseline at post‐boost 1 and post‐boost 2 (Figure S6e,f).These results suggest that TIGIT+DNAM+ LN Tfh and IL‐21R+ LN B cells play key roles in the magnitude of vaccine‐induced Ab responses.

### 2.6 Transcriptional signatures of IL‐21 induced immunomodulation

**FIGURE 4:**
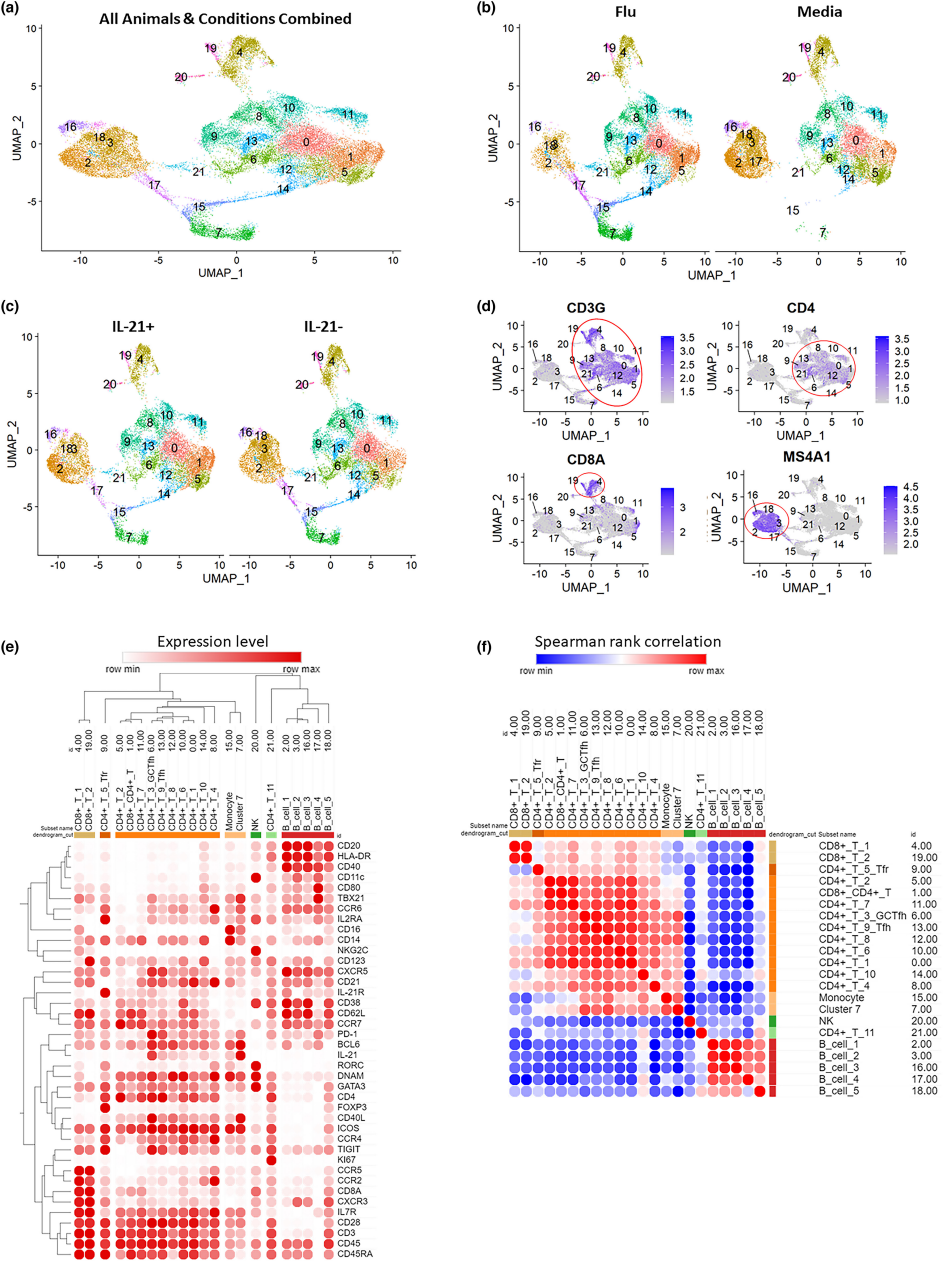
UMAP cluster identification by gene expression: (a), Uniform Manifold Approximation and Projection (UMAP) dimensional reduction of Day 14 post‐B1 flu‐stimulated and unstimulated media ex‐vivo samples from two animals in the old SIV+IL‐21+ and two animals from the old SIV+IL‐21− groups. Clusters were identified in an unsupervised manner via shared nearest neighbor modularity optimization‐based Louvain clustering algorithm. (b) Visualization of UMAP dimension reduction and clusters present in flu stimulated (left) and the unstimulated media ex‐vivo samples (right). (c) Visualization of UMAP dimension reduction and clusters present in old SIV+IL‐21+ (left) and old SIV+IL‐21− (right). (d) Feature UMAP plots showing gene expression of CD3, CD4, CD8a, and CD20 (MS4A) for identification of cell types. (e) Further cluster cell‐type identification with a panel of selected/canonical genes displayed as relative average expression levels for each cluster normalized to row min and max. Hierarchical clustering was performed on columns and rows, with a dendrogram cut to separate out the major cell types identified. (f) Spearman rank correlation similarity matrix based on the average gene expression of all genes included in part E to identify most similar subsets.

**FIGURE 5:**
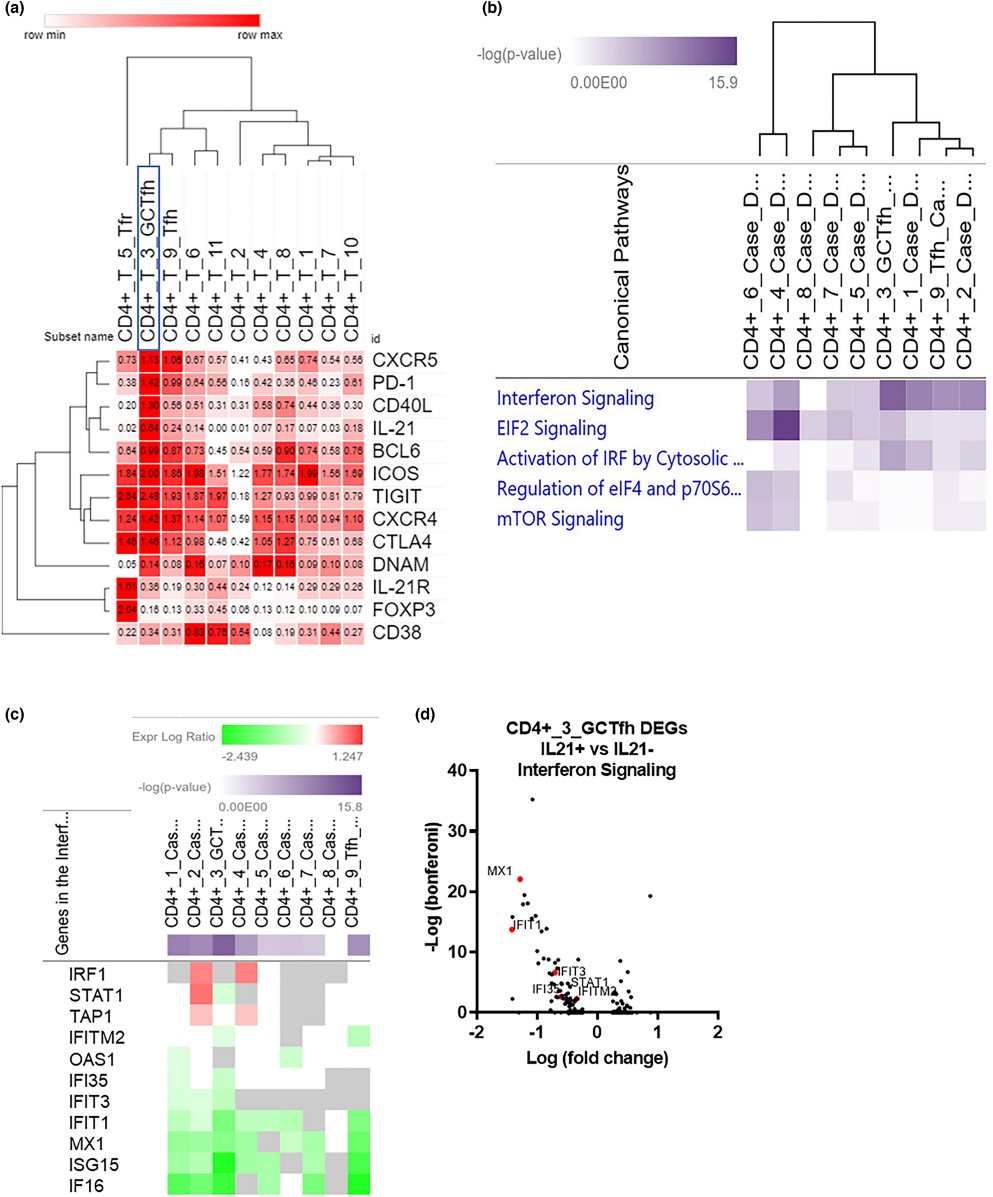
IL‐21 reduces interferon signaling in Tfh, GC Tfh, and CD4 T cell clusters: (a) Selected genes displayed as relative average expression levels for each CD4 T‐cell cluster normalized to row min and max. Hierarchical clustering was performed on columns and rows. (b) Canonical pathway analysis of differentially expressed genes (DEGs) in each CD4 T cell cluster between old SIV+IL‐21+ and old SIV+IL‐21− unstimulated media ex‐vivo samples. Significant canonical pathways are shaded from light purple to dark purple based on the −log (*p* value). (c) Heatmaps of the expression log ratio (down = green, up = red) of significant DEGs present in the interferon signaling canonical pathways for all CD4 T‐cell clusters. (d) Volcano plots of significant DEGs that are downregulated (highlighted in red) from the IFN signaling pathway detected in GC‐Tfh cluster 9.

To comprehensively assess IL‐21 immunomodulation of the cellular and humoral immune response within draining LNs, we performed single‐cell 5′ gene expression profiling. Dimensionality reduction of 32,000 total cells from both flu‐stimulated and ‐unstimulated (ex‐vivo) LN cells of two IL‐21‐treated and two IL‐21‐untreated animals by UMAP identified 21 clusters of cells (Figure 4a–d) and the relative proportion of clusters did not differ between IL‐21 treated and untreated animals. Next, we plotted a relative gene expression heatmap using a list of selected genes known to be associated with phenotypic characterization of T cells, B cells, monocytes and NK cells and performed hierarchical clustering so that similar subsets would be clustered together for cell type identification (Figure 4e). We identified 1 cluster of monocytes (Cluster 15) as Lin‐CD14+CD16+, 1 cluster of NK cells (Cluster 20) as Lin‐CD8a+NKG2C+, 5 clusters of B cells as Lin‐CD20+HLA‐DR+CD40+ (Clusters 2, 3, 16, 17, and 18), 2 clusters of CD8+ T cells as Lin‐CD3+CD8+ (clusters 4 and 19), 1 cluster of CD4+CD8+ T cells as Lin‐CD3+CD4+CD8+ (cluster 1), and 11 clusters of CD4+ T cells as Lin‐CD3+CD4+ (Figure 4e: Clusters 0, 5, 6, 8, 9, 10, 11, 12, 13, 14, and 21). Within CD4 T‐cell clusters, Cluster 9 was identified as mix of T follicular regulatory cells (Tfr) and T regulatory (Treg) cells based on the expression of CD25 and FOXP3 (Figure 4e). CD4 Clusters 6 and 13 were identified as GC Tfh and LN Tfh, respectively, based on their high expression of BCL6, CXCR5, PD‐1, IL‐21, and CD40L (Figure 4e). Within CD4 T‐cell subsets we focused our analysis on LN Tfh and GC Tfh cells and observed that GC Tfh and LN Tfh cells express high levels of TIGIT relative to other CD4 T‐cell subsets, except Tfr/Treg cells (Cluster 9) which also express high levels of TIGIT as expected (Figure 4e). The expression of DNAM on GC Tfh (Cluster 6) was high relative to other CD4 T‐cell subsets, while DNAM on LN Tfh (Cluster 13) was similar to other CD4 T‐cell subsets, reflecting our observation that TIGIT expression is increased in LN Tfh and GC Tfh, and the co‐expression of TIGIT and DNAM is higher among LN Tfh/GCTfh relative to CD4 naïve and non‐Tfh memory cells (Figure 4e). Next, we performed spearman rank correlation based on the expression of all the genes included in Figure 5e to create a similarity matrix to help identify which cell types were most similar (Figure 4f). Overall, using relative gene expression we were able to identify monocyte, NK cell, B cell, CD8 and CD4 T‐cell subsets, including GC Tfh and LN‐Tfh clusters.

### 2.7 Post‐B1 LN CD4 T cells and GC Tfh from IL‐21‐treated animals have decreased interferon signaling

After subset identification, we sought to explore biological functions and pathways to identify potential mechanisms by which IL‐21 modulates LN Tfh/GC Tfh gene expression to support vaccine‐induced Ab responses. First, differentially expressed genes (DEGs) between IL‐21‐treated versus ‐untreated from the unstimulated ex‐vivo condition was analyzed using Qiagen ingenuity pathway analysis (IPA). Based on the relative gene expression of BCL6, PD‐1, CXCR5, and TIGIT in each CD4 T‐cell cluster we were able to identify LN Tfh and GC Tfh. GC Tfh showed higher relative gene expression of IL21, CD40L, and DNAM than Tfh (Figure 5a). Next, we performed canonical pathway analysis using the DEGs from each CD4 T‐cell cluster and plotted a heatmap of the most significant pathways differentially regulated in IL‐21‐treated animals (Figure 5b). The most significant differentially regulated pathway in LN Tfh and GC Tfh was the interferon signaling pathway, leading us to investigate the differential expression of genes belonging to the interferon pathway (Figure 5c,d). GC Tfh and LN Tfh clusters both had significant downregulation of IFITM2, IFIT1, MX1, ISG15, and IFI6 in IL‐21‐treated animals compared to untreated (Figure 5c). Additionally, GC Tfh had significant downregulation of IFIT3, IFI35, and STAT1 in IL‐21‐treated versus untreated (Figure 5c,d). Together these results show that IL‐21 immunotherapy in the context of flu vaccination lowers Interferon signaling in LN CD4 T‐cell subsets and of particular interest, in LN Tfh and GC Tfh cells, suggesting that these cells have lower inflammatory signaling while maintaining B cell help properties as evident by increased Ab responses in IL‐21‐treated animals.

### 2.8 Post‐B1 LN B cells from IL‐21‐treated animals have decreased interferon signaling and enhanced B‐cell development and antigen presentation pathways

Next, using the same approach as applied to LN Tfh/GC‐Tfh cells, we aimed to further characterize alterations in B‐cell transcriptional profiles and biological pathways as a result of IL21 treatment. Across all B cells, the most significant and relevant pathways that differ between IL‐21‐treated compared to untreated were interferon signaling (downregulated), activation of IRF by cytosolic pattern recognition receptors (downregulated), antigen presentation (upregulated), B‐cell development (upregulated), Th1 (upregulated), Th2 (upregulated), and IL‐4 (upregulated) signaling pathways (Figure S7).

**FIGURE 6:**
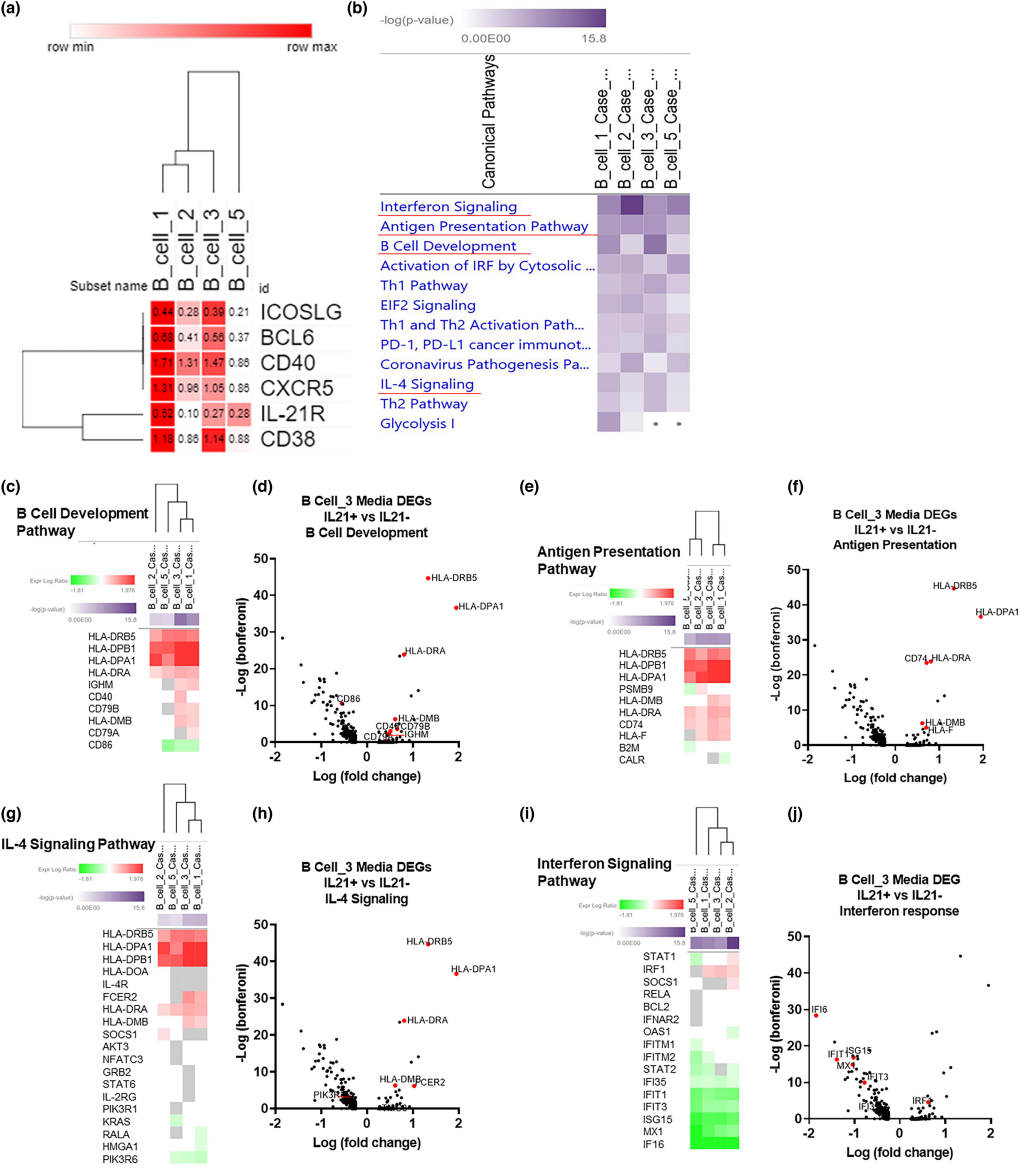
IL‐21 enhances B‐cell development, antigen presentation, and IL‐4 signaling: (a) Selected/canonical genes displayed as relative average expression levels for each B cell cluster normalized to row min and max. (b) Canonical pathway analysis of differentially expressed genes (DEGs) in each B‐cell cluster between old SIV+IL‐21+ and old SIV+IL‐21− unstimulated media ex‐vivo samples. Nonsignificant canonical pathway detection is indicated by a dot; all other canonical pathways are significant and shaded from light purple to dark purple based on the −log (*p* value). (c, e, j, i) Heatmaps of the expression log ratio (down = green, up = red) of significant DEGs present in each indicated canonical pathway for all B‐cell clusters. (d, f, h, j) Volcano plots of significant DEGs (highlighted in red) from the B‐cell development pathway detected in B‐cell cluster 3.

Next, we analyzed the B‐cell clusters identify pathways that are upregulated or downregulated as a result of IL‐21 immunotherapy. DEGs from B‐cell cluster 4 were not generated due to low cell numbers. To better compare LN B cell subsets, we created a heatmap comparing relative gene expression of key B cell genes (Figure 6a). B‐cell clusters 1 and 3 have higher expression of CXCR5, CD40, IL‐21R, CD38, BCL6, and ICOSLG, suggesting that these clusters more closely identify activated GC B cells compared to B‐cell clusters 2 and 5 (Figure 6a). Next, we performed comparison analysis of the DEG‐generated biological pathways for each of the B‐cell clusters and plotted them based on their level of significance (Figure 6b). Similar to the DEG pathway analysis for all cells, the most significant differentially regulated pathways in IL‐21‐treated versus ‐untreated animals across B‐cell subsets included interferon signaling (downregulated), antigen presentation (upregulated), B‐cell development (upregulated) as well as IL‐4 signaling (upregulated) among others (Figure 6b). Of note, B‐cell development pathways were most significant in B‐cell clusters 1 and 3, which as previously mentioned, also have relatively higher expression of GC B cell markers (Figure S8a,b). Thus, we further investigated differentially expressed pathways for B‐cell clusters 1 and 3 (Figure S8a,b). Interestingly, the most significant differentially regulated pathway in B cell cluster 1 between IL‐21‐treated and ‐untreated was interferon signaling (downregulated) followed by B‐cell development (upregulated), antigen presentation (upregulated), and glycolysis (downregulated) (Figure S8a). In B‐cell cluster 3, it was B‐cell development (upregulated) followed by interferon signaling (downregulated), and antigen presentation (upregulated) (Figure S8b). These results show that the most prominent pathways which are differentially regulated by IL‐21 immunotherapy are shared across B‐cell subsets. Next, we investigated the DEGs within B‐cell development (Figure 6c), antigen presentation (Figure 6e), IL‐4 signaling (Figure 6g), and interferon signaling pathways (Figure 6i) across B‐cell subsets. Additionally, we plotted differentially expressed genes from B‐cell cluster 3 as a representative visualization of the genes involved in each pathway and their significant differential expression (Figure 6d,f,h,j). Of note, HLA‐DRB5, HLA‐DPB1, HLA‐DPA1 and HLA‐DRA were upregulated in all B‐cell clusters (Figure 6c–h). Within the B cell development pathway, B‐cell clusters 1 and 3 had the most DEGs, with significant upregulation of IGHM, CD79B and HLA‐DMB (Figure 6c). Additionally, B‐cell cluster 3 had significant upregulation of CD40, while B‐cell cluster 1 had upregulation of CD79A (Figure 6c). CD86 was downregulated in B‐cell clusters 1, 3, and 5 but not 2 (Figure 6c). Within the antigen presentation pathway, HLA‐DRA and CD74 were upregulated in all B cell clusters, while HLA‐DMB was upregulated in only B‐cell clusters 1 and 3 (Figure 6e). In the IL‐4 signaling pathway, B‐cell clusters 1 and 3 had upregulation of FCER2 and HLA‐DMB in addition to upregulation of the genes overlapping with B‐cell development and antigen presentation (Figure 6g). The Interferon signaling pathway showed robust downregulation, with all B‐cell clusters having downregulated IFI35, IFIT1, IFIT3, ISG15, MX1, and IFI6 transcripts (Figure 6i). STAT2 was downregulated in B‐cell clusters 1, 2 and 5 (Figure 6i). Interestingly, IRF1 was upregulated in B‐cell clusters 1, 3 and 2 (Figure 6i). Here we show that IL‐21 immunotherapy in the context of flu vaccination upregulates genes involved with B‐cell development, antigen presentation and IL‐4 signaling and downregulated interferon signaling across B‐cell subsets, providing evidence for mechanisms that explain the enhanced vaccine‐induced Ab responses in IL‐21‐treated animals.

## 3 DISCUSSION

In the current study, we aimed to further investigate our previous findings that IL‐21 improves the seasonal flu vaccine Ab response in old SIV+ rhesus macaques (32) by studying IL‐21‐induced immunomodulation of Tfh and B‐cell subsets within draining LNs. We recently reported an association between increased frequencies of TIGIT expressing pTfh detected in IL‐21‐treated animals with increased vaccine‐induced Ab response (Kvistad et al., 2021). TIGIT and DNAM among others, which are expressed on T cells and NK cells and share a common ligand CD155, expressed on APCs, are members of a potent innate and adaptive immunoregulatory axis. It was recently shown that TIGIT expressing pTfh in circulation have superior B‐cell helper properties, fostering greater Ab production than their TIGIT‐ counterparts (Godefroy et al., 2015). Further, it was demonstrated that blocking TIGIT abrogates pTfh‐derived B cell helper functions, implicating TIGIT as a functional receptor in Tfh/B‐cell interactions which take place in LN GC reactions (Godefroy et al., 2015). We found that the expressions of TIGIT and DNAM was highly varied between CD4 subsets. Interestingly, LN GC Tfh cells were notably distinct from LN naïve, non‐Tfh memory, and Tfh cells with a predominant DNAM−TIGIT+ phenotype followed by DNAM+TIGIT+ cells. Flu‐stimulated AIM+ LN Tfh and AIM+ LN GC Tfh were enriched for DNAM−TIGIT+ and DNAM+TIGIT+ populations compared to unstimulated ex‐vivo total LN Tfh and LN GC Tfh. These data indicate that activated flu‐specific AIM+ LN Tfh and GC Tfh shift phenotype away from DNAM−TIGIT− and DNAM+TIGIT− toward DNAM−TIGIT+ and DNAM+TIGIT+. It has been reported that TIGIT is upregulated on T cells following activation, as a mechanism to inhibit hyperactivation (Linterman et al., 2010), supporting our observation of increased TIGIT expression on AIM+ LN Tfh and GC Tfh populations. Our data further showed a preferential expression of IL‐21R on DNAM expressing cells implying that DNAM expressing Tfh subsets may be highly responsive to IL‐21 immunotherapy.

Considering that DNAM is an activating receptor and TIGIT is an inhibitory receptor, we hypothesize that LN CD4 naïve and non‐Tfh memory cells, which have high DNAM single expression are poised to become activated following cognate interaction with APCs, while LN Tfh and particularly GC Tfh, which display substantial TIGIT expression alongside DNAM are biased for a more balanced activation state. Additionally, TIGIT may be playing a key role in regulating activating signals transmitted through DNAM, culminating in a measured activation state which is fine‐tuned for B‐cell help. We found a DNAM/TIGIT MFI ratio of ~3:1 on both naïve and non‐Tfh DNAM+TIGIT+ memory CD4 T cells, while DNAM+TIGIT+ Tfh had a ratio of ~2:1 and DNAM+TIGIT+ GC Tfh had a ratio of ~1:1. These results describe a possible transition from high to low DNAM/TIGIT MFI ratios during differentiation from CD4 naïve to LN Tfh and GC Tfh, supporting our hypothesis on the role of TIGIT and DNAM in regulating the activation/inhibitory signals that LN Tfh and GC Tfh cells receive. Furthermore, DNAM plays a dominant role during CD4 naïve/memory activation as well as early human tonsillar Tfh differentiation, demonstrating that as CD4 T cells differentiate toward Tfh and GC Tfh, TIGIT expression is greatly increased (Yasutomi et al., 2022). Moreover, they showed that excessive DNAM signaling, and associated activation is detrimental to GC Tfh integrity and that TIGIT may be required to insulate against DNAM‐dependent activation signals (Yasutomi et al., 2022).

Given the high IL‐21R expression among DNAM+TIGIT+ LN Tfh/GC Tfh, we investigated DNAM+TIGIT+ co‐expressing Tfh subsets between IL‐21‐treated and controls. We found that DNAM+TIGIT+ LN Tfh and post‐B1 and post‐B2 GC Tfh correlated with the magnitude of HAI titer, density of Tfh/follicle per area mm^2^ and the frequency of Day 14 post‐B1 IL‐21R+ B cells. Moreover, frequency of unstimulated ex‐vivo Day 14 post‐B1 IL‐21R+ B cells correlated with post‐B1 and study endpoint HAI titer FC. These findings underscore the importance of IL‐21R expression on B cells which is required for maximal BCL6 expression (Linterman et al., 2010), and is critical for T‐dependent B‐cell responses and GC reactions that lead to robust and long‐lasting protective humoral immunity following infection or vaccination (Tangye & Ma, 2020). Together, the data suggest that DNAM+TIGIT+ LN Tfh represent a subset which is more responsive to IL‐21 immunotherapy, provides strong help to B cells and may directly or indirectly increase B cell IL‐21R expression, fostering improved GC reactions and increased vaccine‐induced Ab responses.

Transcriptional profiles of Tfh and B cells from post‐B1 draining LNs by single‐cell analysis demonstrated that IL‐21‐treated animals had significantly upregulated expression of B‐cell development, antigen presentation, and IL‐4 signaling pathways as well as downregulation of the interferon signaling pathway. Significantly upregulated genes in the B‐cell development pathway across the B‐cell clusters include *CD79A*, *CD79B*, *HLA‐DRB5*, *HLA‐DPB1*, *HLA‐DPA1*, *HLA‐DRA*, *IGHM*, *HLA‐DMB*, and *CD40*. In mature B cells, CD79a/b are co‐receptors that associate with surface Ig including IgM and form the BCR complex. In accordance with this *IGHM* was also significantly upregulated in IL‐21‐treated animals and implies increased activation and survival of B cells through BCR antigen recognition or IL‐4 signaling (Katikaneni & Jin, 2019; Treanor, 2012; Wen et al., 2019). *HLA‐DRB5*, *HLA‐DPB1*, *HLA‐DPA1*, *HLA‐DRA*, and *HLA‐DMB* are components of the Class II major histocompatibility complex (MHC) and belong to the B cell development, antigen presentation and IL‐4 signaling pathways, all of which were upregulated in IL‐21‐treated animals. Increased expression of MHC class II alongside increased *CD40* gene expression indicates enhanced antigen presentation to cognate CD4 T cells along with increased activation, proliferation and differentiation of B cells may improve the antibody responses (Katikaneni & Jin, 2019).

An important effect of IL‐21 was to dampen IFN mediated inflammation, as exemplified by significant downregulation of genes associated with interferon signaling pathways, for example, *IFITM2*, *IFIT1*, *MX1*, *ISG15*, and *IFI6* in Tfh and GC Tfh, which also downregulated *IFIT3*, *IFI35*, and *STAT1* Unlike acute infection where induction of interferon type 1 and the activation of interferon‐stimulated genes (ISGs) facilitate DC, B‐, and T‐cell effector functions, sustained IFN‐I expression during chronic infection drives deleterious immunomodulatory effects (Murira & Lamarre, 2016). with immune activation, T‐cell exhaustion, and cell death (Fraietta et al., 2013; Hardy et al., 2013; Rout et al., 2022). LN Tfh from SIV‐infected NHPs, display an altered transcriptional signature composed of upregulated ISGs (*IFI27*, *IFI44*, *IFI6*, and *MX1*) compared to Tfh from SIV‐ lymph nodes (Petrovas et al., 2012; Vella et al., 2017).We have previously reported a predictive gene‐expression signature composed of Type I interferon‐induced genes in flu‐specific B cells from aging PWH compared to PWoH (de Armas et al., 2019). Together, the significant downregulation of ISGs in Tfh, GC Tfh and B cells from IL‐21‐treated animals provides a mechanistic explanation for the role of IL‐21 in reversing HIV/SIV‐associated Tfh and B‐cell dysfunction leading to enhance vaccine‐induced Ab responses.

These results indicate that IL‐21 immunotherapy promotes an anti‐inflammatory LN GC microenvironment and may bias GC Tfh cells towards Tfh2 type, supporting strong flu vaccine‐induced Ab production. Furthermore, the IL‐21‐induced reduction of IFN signaling observed in the present study may help to explain our previous findings of acutely SIV‐infected RMs treated with IL‐21 displaying an increase in intestinal Th17 cell, along with decrease in T‐cell immune activation and systemic inflammation during chronic infection (Pallikkuth et al., 2011; Pallikkuth et al., 2013).

In conclusion, our study supports the immunomodulatory effect of IL‐21 in aging and SIV infection by altering the expression of immune check point molecules on Tfh cells along with an overall lowering of IFN signaling in both Tfh and B cells in LN leading to enhanced Ab responses. This observation is supported by the strong positive relationship of DNAM+TIGIT+ Tfh with vaccine responses, IL‐21R+ B cells, and GC activity. Further we contend that IL‐21 immunomodulation inhibits deleterious SIV associated immune activation and inflammation by significantly downregulating interferon‐induced genes in both Tfh and B cells in the LN concomitantly with upregulation of genes associated with B cell development, antigen presentation and IL‐4 signaling pathways. Future studies are warranted to explore the overall benefit of IL‐21 immunotherapy on mucosal lung immunity and protection against acquiring the lung infection in aging and SIV. Nevertheless, this study provides mechanistic insight into IL‐21‐mediated immunomodulation of Tfh and B cells that results in improved Ab responses to flu vaccination in an aging SIV+ rhesus macaque model.

## 4 MATERIALS AND METHODS

### 4.1 Study animals and immunization

As previously published (Kvistad et al., 2021), Indian RMs with no history of recent/current infection or vaccination housed at the New Iberia Research Center (NIRC) at the University of Louisiana at Lafayette were used in this study. For this study, 12 old (mean: 21 years, range: 3.8 years) female (*n* = 11) and male (*n* = 1) animals were enrolled, divided into subgroups old SIV‐infected IL‐21–IgFc–untreated (*n* = 4), and old SIV‐infected IL‐21–IgFc–treated (*n* = 8). All animals were infected with 200 median tissue culture infective dose (TCID_50_) of SIVmac239‐*nef‐stop* (Villinger, 2020) administered by i.v. At 12 weeks post‐infection and the remainder of the study, animals were treated with ART consisting of tenofovir (TFV; 20 mg/kg/day) combined with emtricitabine (FTC; 30 mg/kg/day) s.c. (both from Carbosynth) and raltegravir (L870,812 donated by Merck Sharp & Dohme Corp.) Three months after ART initiation, all animals were vaccinated with the trivalent 2015–2016 seasonal flu vaccination (Afluria vaccine, bioCSL) carrying 15 μg each of H1N1, H3N2, and B antigens in a prime/boost/boost strategy at 3‐month intervals. Vaccine was administered intramuscularly in the deltoid region after splitting the dose in to two and administer at both left and right deltoid. The axillary lymph nodes were collected on Day 14 post each vaccine dose. For each of the three vaccine doses, IL‐21‐IgFc (NHP Immune Reagents Resource) ‐treated animals received 50 μg/kg subcutaneously in 3 doses: (1) on Day 2 before each vaccination to prime immune cells, (2) concurrently with the vaccine and collocated to the site of vaccination, and (3) 7 days after vaccination. Similar to vaccine, IL‐21‐IgFc was also split in two for right and left deltoid administration.

### 4.2 Samples

Blood samples collected at Day 0 (before vaccination), Day 14, Day 42 after each vaccine dose, and at Day 84 post‐B2 and processed for PBMC, serum, and plasma. Draining LNs biopsies were performed at Day 14 after each vaccine dose (Kvistad et al., 2021), homogenized and passed through a 70 mm cell strainer for mechanical isolation of lymphocytes and cryopreserved until time of assay.

### 4.3 Flu antibody response

The serum antibody response to flu vaccination was determined by HAI titers using the chicken red blood cells as previously described (George et al., 2015).

### 4.4 Preparation of samples scRNAseq and flow cytometry

Briefly, thawed LNMC or PBMCs were rested for 3 h at 37°C, then stimulated with 2 μg/mL each of pooled H1N1 and H3N2 2015–2016 flu vaccine antigens (inactivated protein antigens were provided by CBER, FDA) together with 1 μg/mL of anti‐CD28 and anti‐CD49d, media with no stimulation as negative control and cells stimulated with 1 μg/mL of staphylococcal enterotoxin B (SEB) included as a positive control. All conditions were incubated for 18 h at 37°C. After incubation, cells were split for either (A) single cell profiling or (B) flow cytometry. For single cell profiling, 1–1.5 × 10^6^ cells from the unstimulated media and flu‐stimulated conditions from Day 14 post‐B1 LNMC samples from two IL‐21‐treated and two ‐untreated animals were used. Cells were stained with live/dead blue (Invitrogen), and resuspended in R10 media for sorting of live cells (BD FACS Aria II). Sorted live cells were then resuspended at a concentration of 10,000 cells/μL and delivered to the sc‐RNA‐Seq analysis for capture and library prep. The remaining PBMC and LNMC cells (2–5 × 10^6^) were stained with live/dead blue (Invitrogen) along with FC‐Block, and stained for surface markers, followed by permeabilization and intracellular staining. Stained cells were fixed and acquired on a Cytek Aurora instrument. Flu‐specific GCTfh/ Tfh cells in LNMC and pTfh cells in PBMC and LNMC were identified using the activation induced marker (AIM) assay using CD25 and CD134 (OX40) as previously described Havenar‐Daughton et al. (2016).

### 4.5 Monoclonal antibodies

The following fluorochrome conjugated macaque specific monoclonal antibodies were used for flow cytometry studies: CD3 (SP34‐2, BD), CD4 (SK3, BD), CD45 (D058‐1283, BD), Ki‐67 (B56, BD), CXCR3 (G025H7, Biolegend), CD25 (BC96, Biolegend), IL‐21R (2G1‐K12, Biolegend), CD27 (O323, Invitrogen), CXCR5 (MU5UBEE, Life Technologies), TIGIT (MBSA43, Thermo), CD8 (SK1, BD), CD20 (2H7, BD), IgG (G18‐145, BD), CD21 (B‐ly4, BD), CD80 (L307, BD), CD14 (MφP9, BD), CCR6 (11A9, BD), OX40 (L106, BD), PD‐1 (EH12.2.H7, Biolegend), HLA‐DR (L243, Biolegend), CCR2 (48,607, R&D), CD123 (7G3, BD), DNAM (11A8, Biolegend), CD16 (3G8, BD), CD11b (ICRF44, Biolegend), IgD (2030‐31, Southern Biotech), Live/Dead (Thermo), CD11c (3.9, BD), CD95 (DX2, BD), CD56 (B159, BD).

### 4.6 Single‐cell RNA sequencing

The 10x Genomics Chromium Next GEM Single Cell 5′ Kit v2 was used to process cell suspensions for 5′ gene expression profiling. The cell suspension volumes were calculated for a target cell recovery of 10,000 cells and loaded on the chromium controller per manufacturer's guidelines. The resultant cDNAs were quantified and assessed on the Agilent Bioanalyzer using the high‐sensitivity DNA kit. The final single cell 5′ libraries were quantified using the Qubit dsDNA high sensitivity and qualitatively evaluated on the Agilent Bioanalyzer using the high‐sensitivity DNA kit. Libraries were sequenced on an Illumina NovaSeq 6000 under recommended settings (PE26x90 with 10 bp dual index) targeting 50,000 PE reads per target cell equivalent.

### 4.7 Custom rhesus macaque reference genome

To create the combined Macaca mulatta and SIV genome, we appended the whole genome sequences and annotation features from Ensemble assembly Mmul_10, annotation release 105 and SIVmac239 (NCBI RefSeq Accession M33262.1) to generate a merged FASTA and GTF suitable for alignment and counting. For single‐cell analysis, a custom reference package was created using this hybrid genome and cellranger's mkref pipeline (10x Genomics Cell Ranger 6.1.2). All 17 supported biotype attributes were selected for during the mkgtf filtering process (Table S1). All other parameters were kept as default.

### 4.8 Expression matrices preprocessing and quality control

The raw scRNAseq fastq files were processed using Cell Ranger from 10X Genomics Technology and aligned to the custom macaque/SIV reference genome. Human ortholog gene names were assigned to the Macaque genes using gprofiler2 (Kolberg et al., 2020). “gprofiler2‐ an R package for gene list functional enrichment analysis and namespace conversion toolset g:Profiler.” All expression matrices were loaded into R version 4.1.2 (R Foundation) using the “Read10X” function from the Seurat library version 4.1.0 (Hao et al., 2021). The Seurat library was also used to perform the analyses.

The expression matrices from eight separate samples (4 subjects X 2 samples; Flu stimulated and Unstimulated) were combined into one Seurat object prior to preprocessing, transformation, and analysis. Pre‐processing removed cells with fewer than 200 genes, greater than 6000 genes, or displaying more than 10% mitochondrial transcripts to filter out low‐quality cells. Each independent sample was down sampled to 4000 randomly selected cells per sample to reduce memory allocation requirements from 70,445 to 32,000 total cells. The “SCTransform” function from the Seurat library was applied to transform the expression matrices via normalization and variance stabilization on each sample (Hafemeister & Satija, 2019).

### 4.9 Integrating and merging expression matrices

To allow comparison across samples, the expression matrices were integrated via the “FindIntegrationAnchors” Seurat function the prior to principal component analysis (PCA) dimension reduction with 50 principal components and subsequent UMAP dimensional reduction. Clusters were identified in an unsupervised manner via shared nearest neighbor modularity optimization‐based Louvain clustering algorithm using a resolution parameter of 0.5.

### 4.10 Manual cell cluster annotation

Cell clusters were manually annotated based on respective canonical marker expression levels. Markers used for the classification of cell types corresponding to clusters include: *CD45*, *CD3*, *CD4*, *CD8a*, *CD20*, *NKG2A*, *BCL6*, *IL‐21*, *IL‐21R*, *ICOS*, *ICOSL*, *CD40*, *CD40L*, *TIGIT*, and *DNAM‐1*.

### 4.11 Statistics

Differential expression analysis was conducted via the “FindMarkers” Seurat function, where the Wilcoxon Rank Sum Test and thresholding criteria of logFC > 1 or <−1 and a Bonferroni‐adjusted *p* < 0.05 to identify differentially expressed genes between and within groups and conditions. Cross sectional flow cytometry and HAI titer data were analyzed using two‐tailed Mann–Whitney *U* tests or by two‐way ANOVA with multiple comparison corrections performed with the two‐stage linear step‐up procedure of Benjamini, Krieger, and Yekutieli. All correlations were performed by two‐tailed Spearman R in GraphPad Prism. All statistical comparison of scRNAseq DEGs, and canonical pathway analysis of B cell and CD4 T‐cell clusters was performed with ingenuity pathway analysis by Qiagen.

### 4.12 scRNAseq visualizations

UMAP visualizations were created using the “DimPlot” and “FeaturePlot” Seurat functions. Volcano plots were created using the ggplot2 library of functions (H. Wickham. ggplot2: Elegant Graphics for Data Analysis. Springer‐Verlag New York, 2016).

## AUTHOR CONTRIBUTIONS

**Suresh Pallikkuth**, designing research studies, analyzing data, initial and final draft preparation. **Daniel Kvistad**, conducting experiments, acquiring data, analyzing data, draft writing. **Tirupataiah Sirupangi**, sample collection and processing. **Alexander Kizhner**, scRNAseq data analysis. **Rajendra Pahwa**, manuscript review and editing. **Mark J. Cameron**, **Brian Richardson**, manuscript review and bioinformatics analysis. **Sion Williams**, **Ana Ayupe**, **Marissa Brooks**, scRNA‐sequencing, manuscript review. **Constantinos Petrovas**, tissue analysis, manuscript review. **Francois Villinger**, animal study design, funding acquisition, manuscript review. **Savita Pahwa**, funding acquisition, research design, final manuscript preparation.

## FUNDING INFORMATION

This study was supported by NIH Grant R01AI123048, to Drs. Savita Pahwa and Francois Villinger; University of Miami CFAR (P30AI073961) laboratory core; Vaccine Research Center, NIAID; and Animal Care, Veterinary and Research staff at the New Iberia Research Center. IL‐21‐IgFc was procured by the Resource for NHP Immune Reagents (NIH grant R24 OD010947 to Dr. Francois Villinger).

## CONFLICT OF INTEREST STATEMENT

The authors have declared that no conflict of interest exists.

## Supporting information


Appendix S1.

## Data Availability

The data that support the findings of this study are available from the corresponding author upon reasonable request.
