# Supplementary material for: IL‐21‐IgFc immunotherapy alters transcriptional landscape of lymph node cells leading to enhanced flu vaccine response in aging and SIV infection
Source: Aging Cell. 2023 Sep 15;22(11):e13984. doi: 10.1111/acel.13984 (PMC10652303; doi:10.1111/acel.13984)
Supplement: Supplementary file 1 — Appendix S1. [file ACEL-22-e13984-s001.docx]

**Supplemental tables**

| **Biotype Parameter Passed to mkgtf** |
| --- |
| --attribute=gene_biotype:protein_coding |
| --attribute=gene_biotype:lincRNA |
| --attribute=gene_biotype:antisense |
| --attribute=gene_biotype:IG_C_gene |
| --attribute=gene_biotype:IG_D_gene |
| --attribute=gene_biotype:IG_J_gene |
| --attribute=gene_biotype:IG_LV_gene |
| --attribute=gene_biotype:IG_V_gene |
| --attribute=gene_biotype:IG_V_pseudogene |
| --attribute=gene_biotype:IG_J_pseudogene |
| --attribute=gene_biotype:IG_C_pseudogene |
| --attribute=gene_biotype:TR_C_gene |
| --attribute=gene_biotype:TR_D_gene |
| --attribute=gene_biotype:TR_J_gene |
| --attribute=gene_biotype:TR_V_gene |
| --attribute=gene_biotype:TR_V_pseudogene |
| --attribute=gene_biotype:TR_J_pseudogene |

**Supplemental table 1.** Biotype for macaque reference genome. 17 biotypes attributes which were selected for during the mkgtf filtering process.

**Supplemental Figures**

**
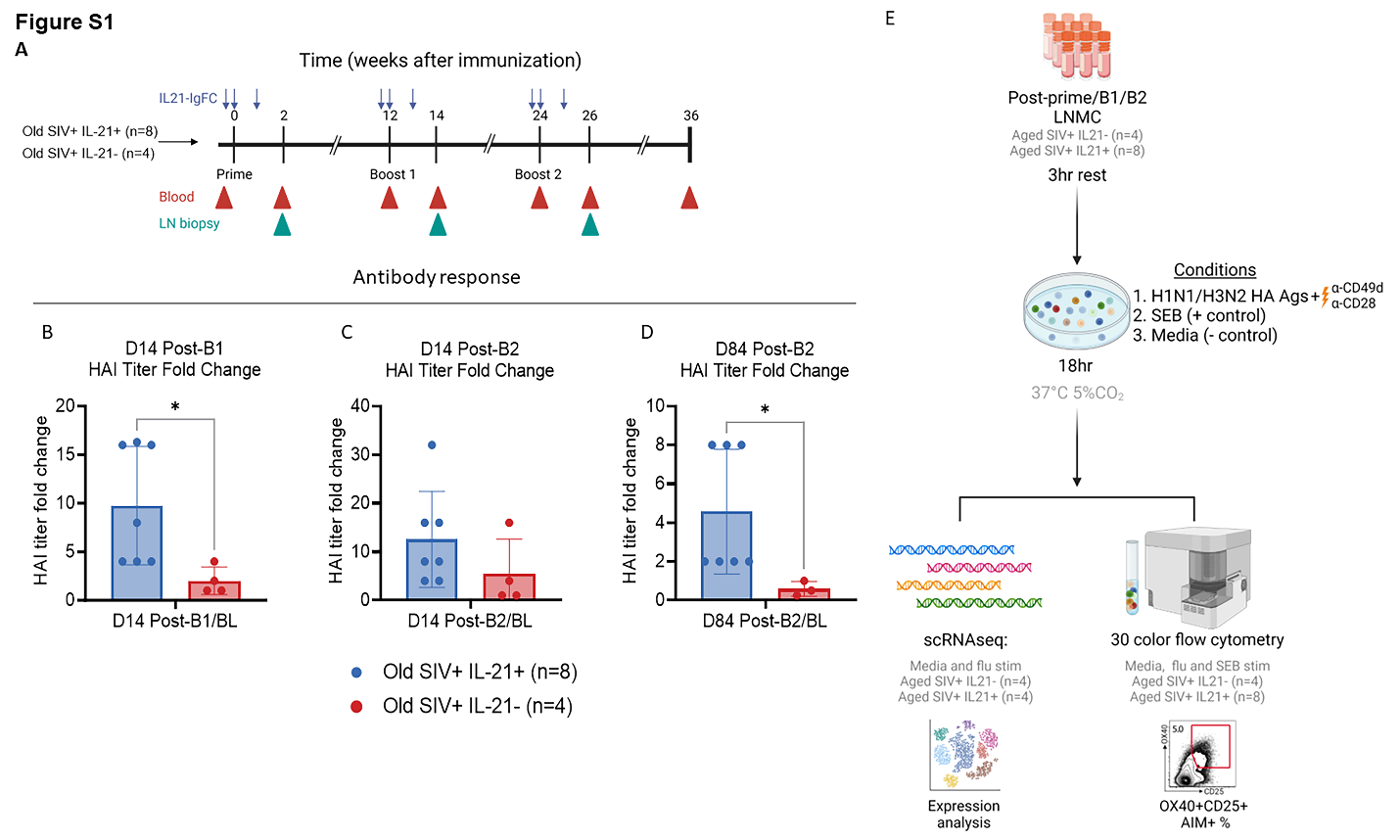
**

**Figure S1. Immunization scheme and analysis strategy:** (A) Eight old SIV+ IL-21 treated and 4 old SIV+ IL-21 untreated animals were infected with SIVmac239 administered at 200 TCID_50_, IV​. ART was initiated 90 days post infection PMPA/FTC/L-000870812. Ninety days post ART-initiation the trivalent 2015-2016 seasonal flu vaccine (Afluria vaccine manufactured by bioCSL with 15 µg each of H1N1, H3N2 and B antigens) was administered intramuscularly in a prime-boost-boost strategy at 3-month intervals. IL-21-IgFc [50µg/kg body weight] was administered subcutaneously in 3 doses: 1) on day -2 before each vaccination at the upcoming vaccination site to prime immune cells, 2) concurrent and co-located to the site of vaccination, and 3) 7 days post vaccination. Red triangles indicate day of blood collection for PBMC, plasma and serum isolation; draining LN collection on D14 post vaccine timepoints is indicated by teal colored triangle. (B) HAI titer fold change from pre-prime baseline to day 14 post B1, (C) post-B2 and (D) day 84 post-B2/study endpoint. (E) Lymph node suspension analysis strategy. Data is displayed as mean ± SEM, blue dots represent old SIV+ IL-21+ animals (due to missing sample from one animal, n=7), while red circles represent old SIV+ IL-21- animals (n=4) two-tailed Mann Whitney tests were performed. *≤0.05; **≤0.01; ***≤0.001; ****≤0.0001.

**
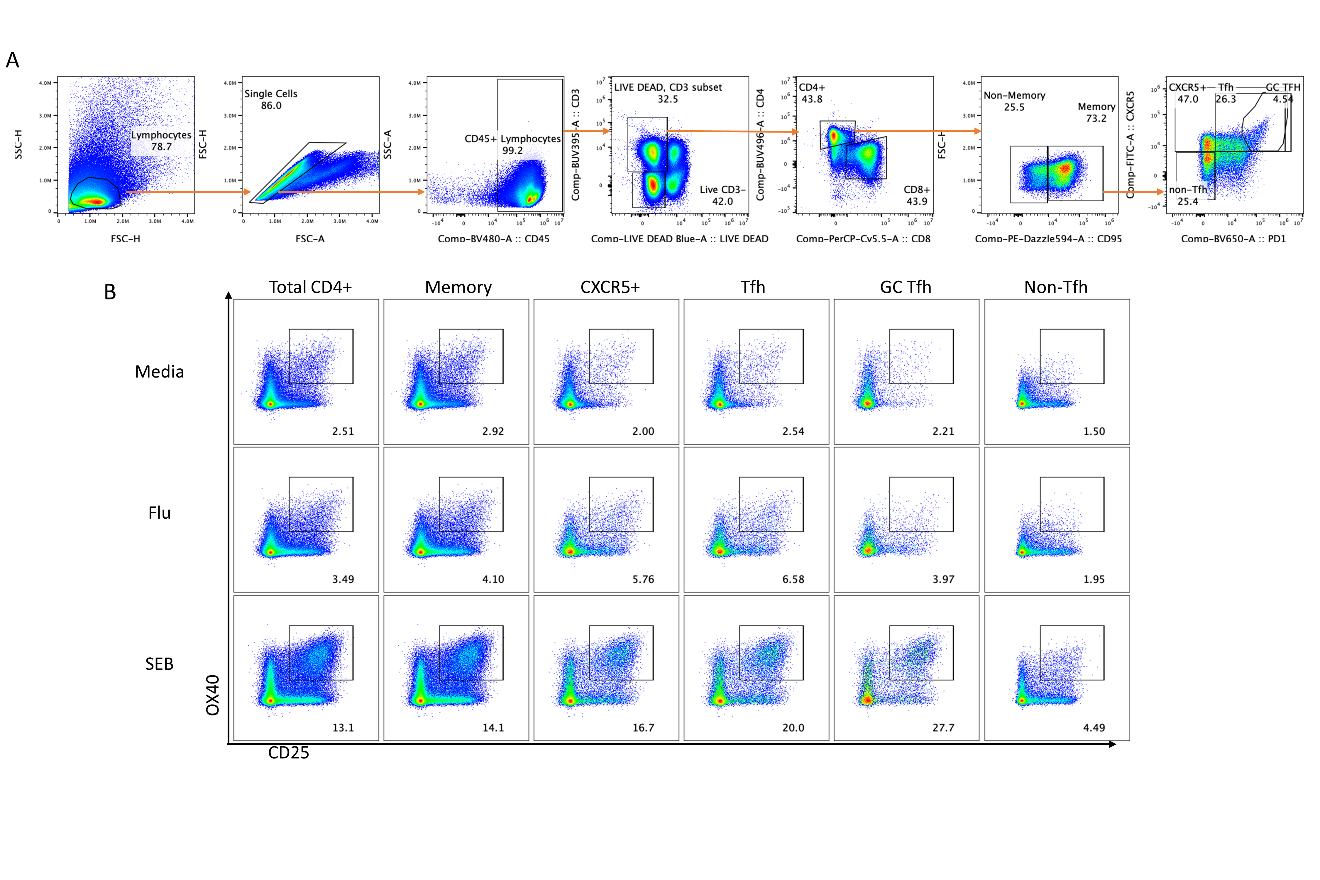
**

**Figure S2:** CD4 T cell and AIM gating strategy.

(A) Gating strategy for subsets of Tfh cells, all of which are CD4+ CD95+ memory cells. Non-Tfh are CXCR5-PD1-, there are total CXCR5+, Tfh (CXCR5+PD1+) and GC Tfh (CXCR5++PD1++). (B) AIM (OX40+CD25+) gating strategy for media, flu and SEB conditions across several CD4 T cell subsets as indicated.


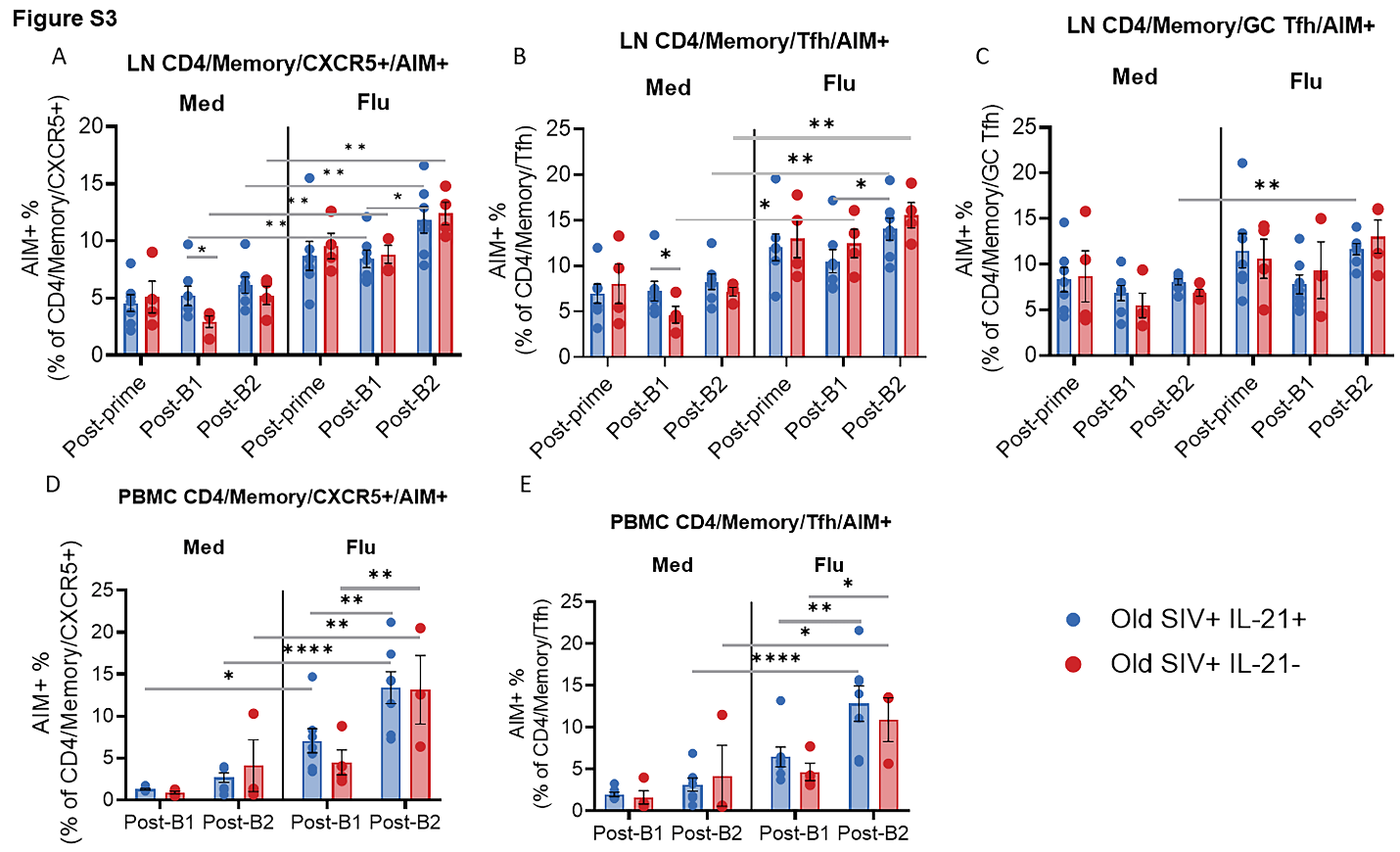


**Figure S3:** LNMC and PBMC AIM+ population frequencies in media and flu-stimulated conditions: Comparison of unstimulated media (ex-vivo) condition vs flu-stimulated condition AIM+ (OX40+CD25+) frequencies of (A) LN CD4+/memory/CXCR5+ T cells, (B) LN Tfh (CD4+/memory/CXCR5+PD1+), (C) LN GC Tfh (CD4+/memory/CXCR5++PD1++), (D) PBMC CD4+/memory/CXCR5+ and (E) PBMC pTfh (CD4+/memory/CXCR5+PD1+). Blue dots represent old SIV+ IL-21+ animals, while red dots represent old SIV+ IL-21- animals. For LNMC samples, old SIV+ IL-21+ n=7 and old SIV+ IL-21- n=4. PBMC samples from day 14 post-prime were not available. In post-B1 PBMC samples, old SIV+ IL-21+ n=7 and old SIV+ IL-21- n=4. In post-B2 PBMC samples, due to sample availability, old SIV+ IL-21+ n=7 and old SIV+ IL-21- n=3. Statistical comparisons were performed by two-way ANOVA with multiple comparison corrections performed with the two-stage linear step-up procedure of Benjamini, Krieger and Yekutieli. *≤0.05; **≤0.01; ***≤0.001; ****≤0.0001.

**
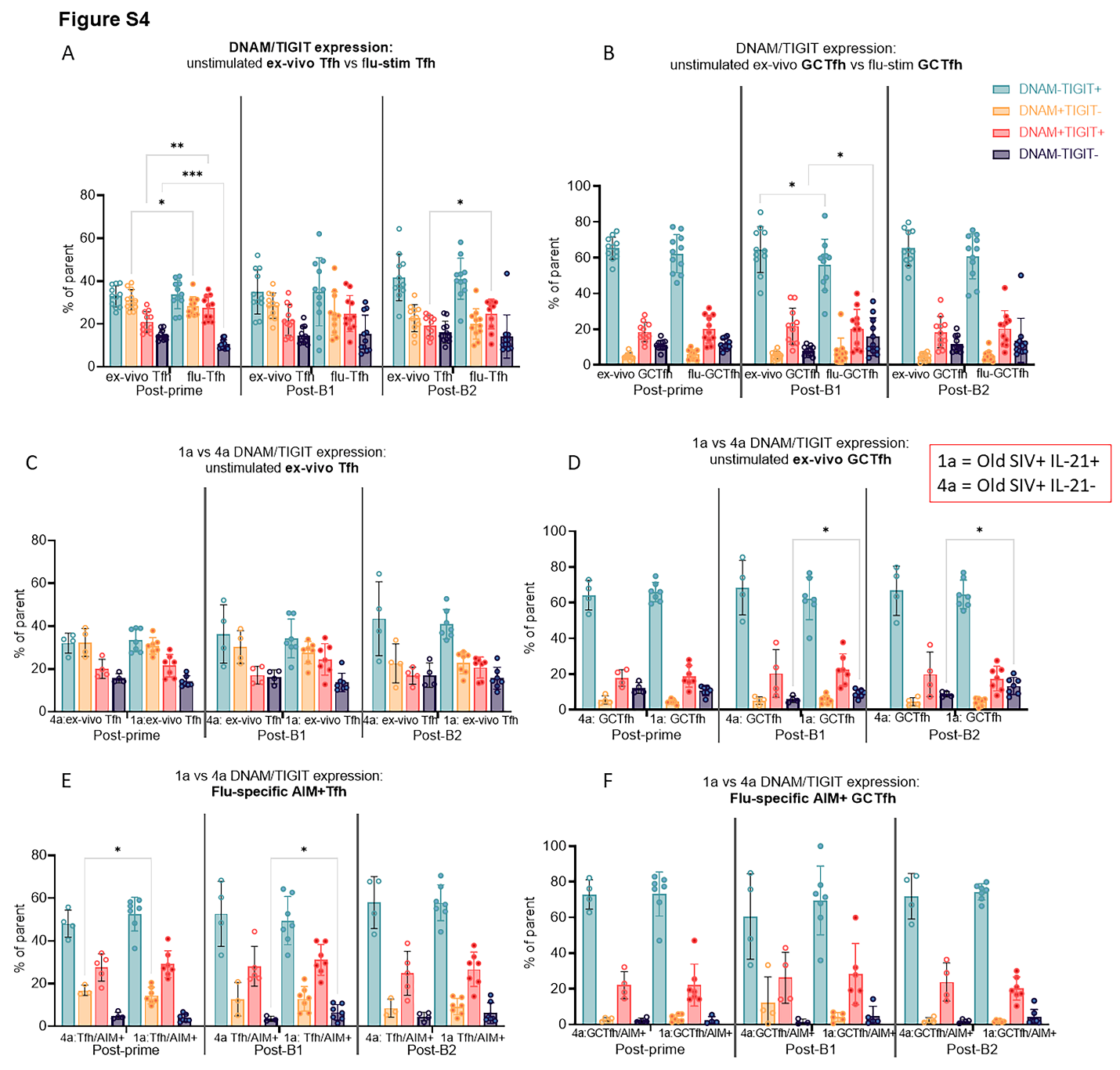
**

**Figure S4:** DNAM and TIGIT expression on ex-vivo Tfh vs total flu-stim Tfh and group comparisons. Frequencies of DNAM-TIGIT+ (teal), DNAM+TIGIT- (yellow), DNAM+TIGIT+ (red) and DNAM-TIGIT- (purple) at all 3 day 14 post-vaccine timepoints compared between (A) ex-vivo (media) total LN Tfh vs flu-stimulated total LN Tfh, (B) ex-vivo total LN GC Tfh vs flu-stimulated total LN GC Tfh, (C) 4a (old SIV+IL-21-) and 1a (old SIV+IL-21+) ex-vivo (media) LN Tfh, (D) 4a (old SIV+IL-21-) and 1a (old SIV+IL-21+) ex-vivo (media) LN GC Tfh, (E) 4a (old SIV+IL-21-) and 1a (old SIV+IL-21+) flu-stimulated AIM+ LN Tfh, and (F) 4a (old SIV+IL-21-) and 1a (old SIV+IL-21+) flu-stimulated AIM+ LN GC Tfh. Data is displayed as mean + SEM. Statistical comparisons were performed by two-way ANOVA with multiple comparison corrections performed with the two-stage linear step-up procedure of Benjamini, Krieger and Yekutieli. *≤0.05; **≤0.01; ***≤0.001; ****≤0.0001.


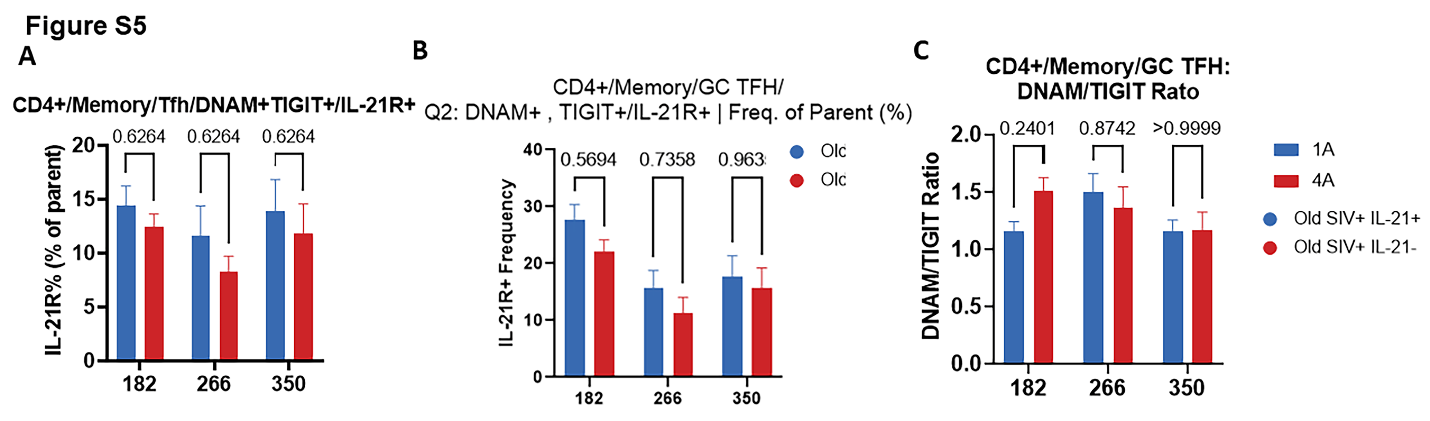


**Figure S5:** Group comparison of IL-21R+ frequency and DNAM/TIGIT ratio on DNAM+TIGIT+ Tfh subsets. (A) IL-21R+ frequency on DNAM+TIGIT+ LN Tfh cells, and (B) DNAM+TIGIT+ LN GC Tfh cells compared at all 3 day 14 post-vaccination timepoints compared between old SIV+ IL-21+ and old SIV+ IL-21- animals. (C) DNAM/TIGIT MFI ratio on DNAM+TIGIT+ LN GC Tfh cells compared between old SIV+ IL-21+ and old SIV+ IL-21- animals. Data is displayed as mean + SEM. Statistical comparisons were performed by two-way ANOVA with multiple comparison corrections performed with the two-stage linear step-up procedure of Benjamini, Krieger and Yekutieli. *≤0.05; **≤0.01; ***≤0.001; ****≤0.0001.


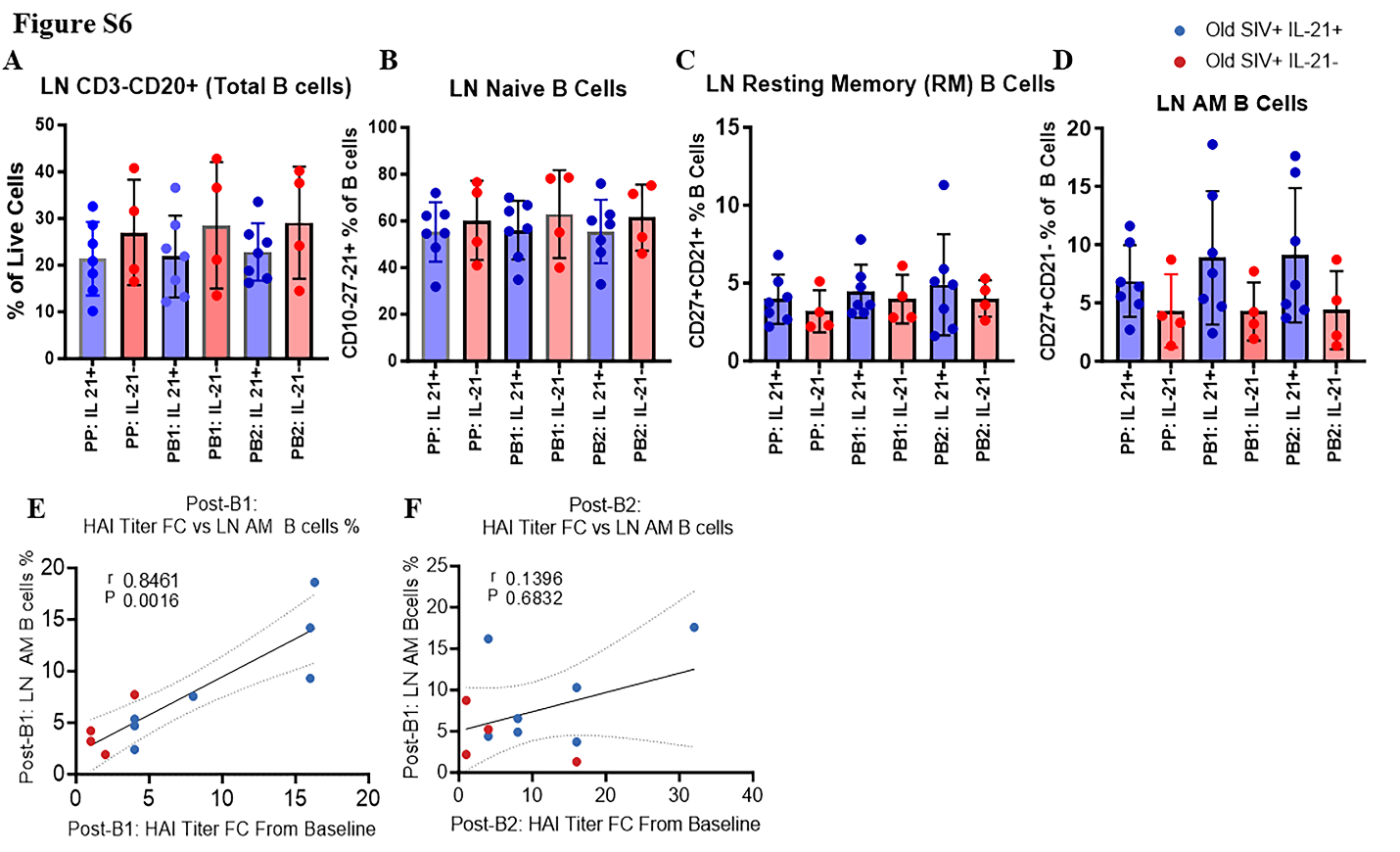


**Figure S6:** B cell subsets in the LN at day 14 post-prime (PP) , day 14 post-boost 1 (PB1) and day 14 post-boost 2 (PB2) were analyzed by flow cytometry. Bar graphs showing frequencies of Total B cells (A), naïve (B), resting memory (C), and activated memory (D) B cells at PP, PB1 and PB2. Correlation between post-B1 (E) and post-B2 (F) LN AM B cells with post-B1 and post-B2 HAI titer fold change from baseline


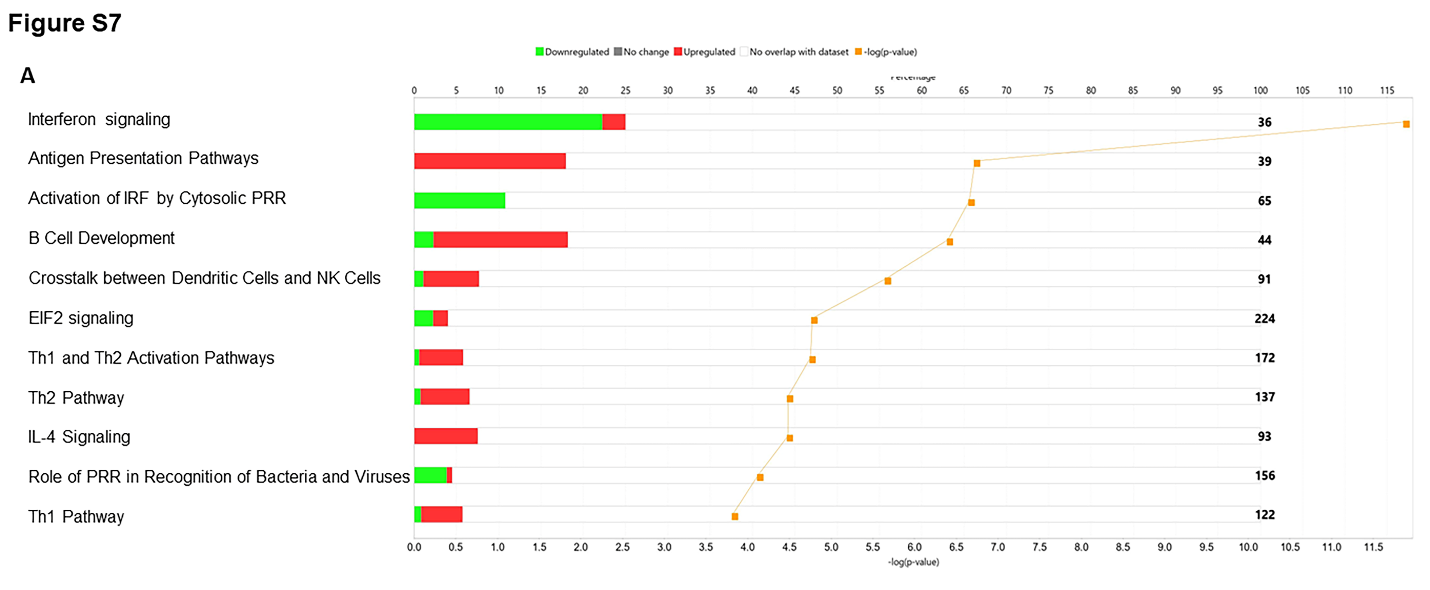


**Figure S:** Pathway analysis of DEGs between IL-21+ and IL-21- for all B cells.

(A) Canonical pathway analysis performed for all DEGs, across all cells, detected between IL-21 treated and untreated animals in the day 14 post-B1 media (ex-vivo) condition. Green indicates downregulated genes, while red indicates upregulated genes. The top X axis represents the percentage of DEGs in our dataset that are part of the indicated canonical pathway. The bottom x axis indicates significance level represented by the orange line and is measured by the -log p value. A -log p value significance threshold of 3.8 was set. Analysis was performed with the Qiagen ingenuity pathway analysis (IPA) program.


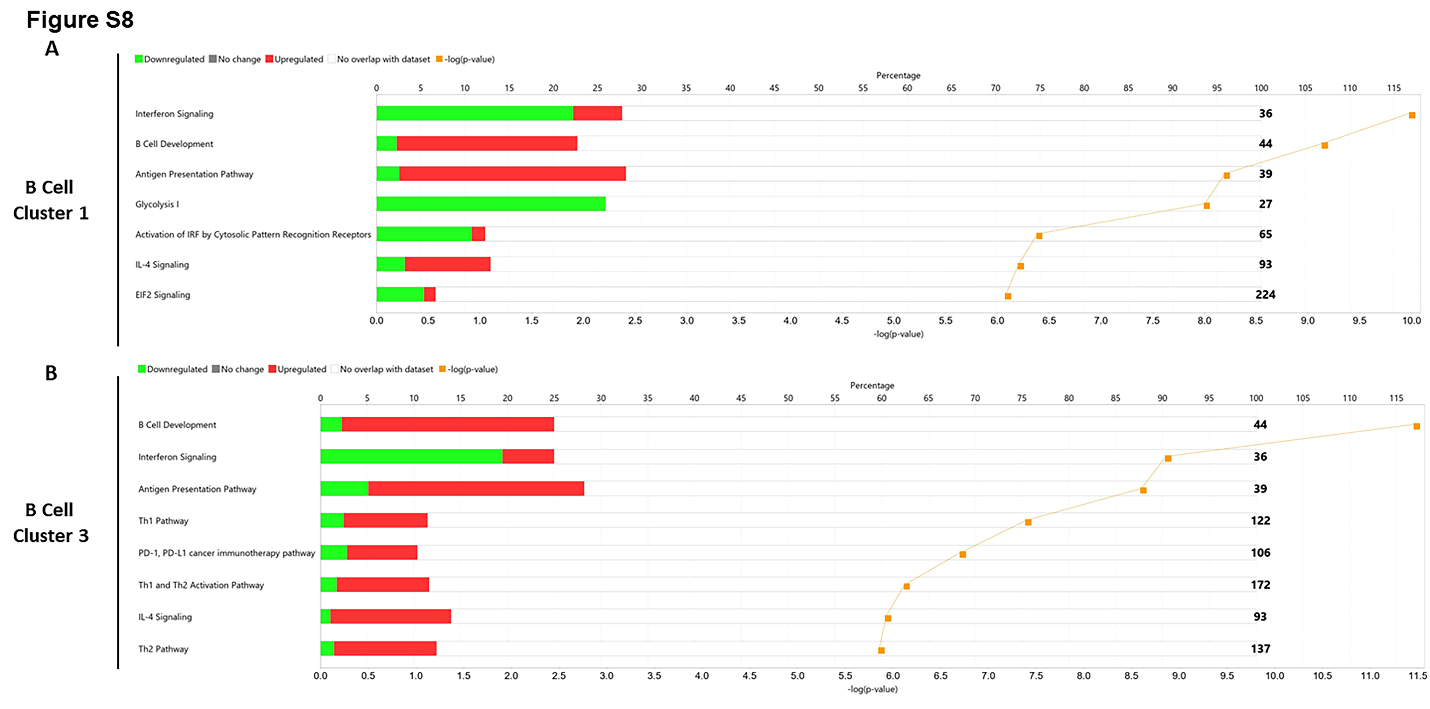


**Figure S8:** B cell clusters 1 & 3 canonical pathway analysis.

Canonical pathway analysis performed for all DEGs, across B cell clusters 1 (A) and 3 (B), detected between IL-21 treated and untreated animals in the day 14 post-B1 media (ex-vivo) condition. Green indicates downregulated genes, while red indicates upregulated genes. The top X axis represents the percentage of DEGs in our dataset that are part of the indicated canonical pathway. The bottom x axis indicates significance level represented by the orange line and is measured by the -log p value. A -log p value significance threshold of 6.2 was set for B cell cluster 1 and a threshold of 5.8 was set for B cell cluster 3 to narrow down the pathways of greatest significance. Analysis was performed with the Qiagen ingenuity pathway analysis (IPA) program.
